# Bayesian Transition Diagnostic Classification Models with Polya-Gamma Augmentation

**DOI:** 10.1017/psy.2025.10031

**Published:** 2025-08-08

**Authors:** Joseph Resch, Samuel Baugh, Hao Duan, James Tang, Matthew J. Madison, Michael Cotterell, Minjeong Jeon

**Affiliations:** 1 Department of Statistics & Data Science, University of California, Los Angeles, CA, USA; 2 Department of Statistics, The Pennsylvania State University, University Park, PA, USA; 3 Department of Education, University of Georgia, Athens, GA, USA; 4 Department of Computer Science, University of Georgia, Athens, GA, USA; 5 Department of Education, University of California, Los Angeles, CA, USA

**Keywords:** diagnostic classification models, Gibbs sampling, intervention effects, Pòlya-gamma augmentation, transition analysis

## Abstract

Diagnostic classification models assume the existence of latent attribute profiles, the possession of which increases the probability of responding correctly to questions requiring the corresponding attributes. Through the use of longitudinally administered exams, the degree to which students are acquiring core attributes over time can be assessed. While past approaches to longitudinal diagnostic classification modeling perform inference on the overall probability of acquiring particular attributes, there is particular interest in the relationship between student progression and student covariates such as intervention effects. To address this need, we propose an integrated Bayesian model for student progression in a longitudinal diagnostic classification modeling framework. Using Pòlya-gamma augmentation with two logistic link functions, we achieve computationally efficient posterior estimation with a conditionally Gibbs sampling procedure. We show that this approach achieves accurate parameter recovery when evaluated using simulated data. We also demonstrate the method on a real-world educational testing data set.

## 1 Introduction

Recent research has developed longitudinal diagnostic classification models (DCMs; Rupp et al., 2010) as psychometric options for diagnostic assessments administered over multiple occasions. These models examine how respondents change in their attribute proficiency statuses over time and have been used to evaluate intervention effects (e.g., Wang et al., 2018). This study focuses on the transition diagnostic classification model (TDCM; Madison & Bradshaw, 2018a) framework, and its direct extension to examine multiple-group differential rates of change in attribute proficiency in a mathematics education research study (Madison & Bradshaw, 2018b). In their analysis, Madison and Bradshaw did not account for covariate effects, which could indicate the differential effectiveness of the instructional intervention. As noted in their discussion, classroom effects were not analyzed because the methodology to do so in a general DCM framework was not available.

To overcome this limitation of the multiple-group TDCM in evaluating interventions, we propose a reformulation of the standard TDCM using hierarchical logistic regressions. The method presented can be thought of in terms of two interrelated components. The first is a binomial logistic measurement model (e.g., log-linear cognitive diagnosis model (LCDM); Henson et al., 2009), forming the relationship between attribute proficiency status to the item response. The second is a multinomial logistic structural model, relating a set of chosen covariates to change in attribute proficiency over time. Thus, the latent transition analysis (LTA) model of the standard TDCM is converted to a full regression formulation. The generalization that is introduced by the proposed reformulation allows for the relaxation of strict conditions that are fundamental to existing DCMs while ensuring proper interpretation and result convergence. At the same time, intervention effects become easy to interpret using log-odds. Most importantly, a highly adjustable covariate structure can be applied to attribute transition trajectories of interest. These benefits in model reformulation serve as the key contribution provided by our proposed model.

Note that the resulting extended TDCM model represents an advanced longitudinal DCM that offers more flexibility than the currently available DCMs. For instance, while DCMs have been adapted for longitudinal assessments, often incorporating a regression-like structure for covariates, most advancements have focused on hidden Markov structures (Liu et al., 2023; Wang et al., 2018; Yamaguchi & Martinez, 2024; Yigit & Douglas, 2021; Zhang & Chang, 2020). Although hidden Markov DCMs are computationally efficient, they can lead to biased conclusions when the underlying Markov assumption is violated (e.g., Pan et al., 2020). More detailed reviews of existing models are provided in Section 2.

The proposed extended TDCM model is accompanied by inference complexity. That is, model inference proceeds over three sets of parameters; the item parameters that link attributes to response probabilities (denoted as 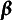 ), the person parameters (i.e., attribute classifications) at each time (denoted as 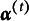 , where *t* indicates discrete time points), and the multinomial regression coefficients for the “progression” of attributes over time (represented as 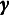 ). To deal with the computational complexity, we propose a Bayesian framework using Pòlya-gamma augmentation (Polson et al., 2013) to be used for both the response and transition logistic models. This procedure allows efficient Gibbs sampling for the TDCM and serves as our second significant contribution. Pòlya-gamma augmentation has been utilized for estimating DCMs. For example, Balamuta et al. (2022) applied the augmentation scheme for the restricted latent class model (RLCM) at a single time point. This was subsequently extended by Jimenez et al. (2023) to multiple time points for the RLCM, although their model does not incorporate a latent transition structure, unlike TDCMs. Further, the multinomial version of Pòlya-gamma augmentation for logistic regressions used in our application has not been applied to longitudinal DCMs, including TDCMs, or in a hierarchical structure for both the item-attribute and transition elements. It is worth stressing that deriving Pòlya-gamma augmentation is neither trivial nor straightforward, given the hierarchical complexity of the proposed TDCM, which includes the item-attribute and transition partitions.

The rest of the article is structured as follows: Section 2 offers a review of the relevant models presented in the literature. Sections 3 and 4 detail the proposed model and its framework for Bayesian inference. Following that, empirical and simulation studies are provided in Sections 5 and 6 with a goal in mind to show the flexibility of the proposed TDCM extension in comparison to the standard TDCM. We conclude our article in Section 7 with a summary and discussion on limitations and avenues for future research.

## 2 Review of related models

In this section, we review models that form the basis for the proposed development. Specifically, we provide both conceptual and technical background on the DCM and the TDCM, which is the motivation of our proposed work. Additionally, we review hidden Markov DCMs in comparison with the proposed TDCM framework.

### 2.1 DCMs

DCMs (Rupp et al., 2010), are psychometric models designed to classify respondents into ordered proficiency categories according to specified categorical latent traits, or attributes. In the dichotomous case, DCMs provide classifications according to each measured attribute, probabilistically placing respondents into one of two groups, typically termed mastery and non-mastery or proficient and non-proficient. Statistically, DCMs are confirmatory and constrained latent class models because 1) they require the specification of a Q-matrix (Tatsuoka, 1983), which delineates the item-attribute alignment; 2) the latent class space is specified a priori as the set of attribute proficiency patterns; and 3) they place constraints on the parameters in a traditional latent class model. Several DCMs have been developed that differ in the way they relate attribute mastery to item response probabilities.

### 2.2 TDCM

To accommodate longitudinal data in a DCM framework, a latent transition model (Collins & Wugalter, 1992) framework can be utilized. The latent transition model is a longitudinal extension of the latent class model, designed to simultaneously classify respondents into latent classes and model their transitions to and from different latent classes over time. Analogous to a DCM being a constrained latent class model, the TDCM; Madison & Bradshaw, 2018a is a constrained latent transition model that classifies respondents into attribute profiles and models their transitions to and from different attribute mastery statuses over time. The TDCM provides measures of student growth on a discrete scale in the form of attribute mastery transitions. In this way, the TDCM supports categorical and criterion-referenced interpretations of growth. On an individual level, growth in the TDCM framework is defined as transitions between different mastery statuses (e.g., non-mastery to mastery). On a group level, growth in the TDCM framework is defined as growth in attribute mastery proportion. For example, if a group went from 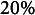 mastery of Standard 1 at time point 1 to 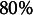 mastery at time point 2, that would be an indication of student learning.

Consider respondent *i* responding to *J* items over *T* testing occasions. In the general form of the TDCM, the probability of the item response vector 
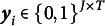
 for respondent *i* is given by 
(1)

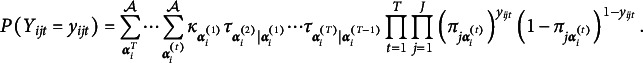



In the above equation, 
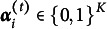
 represents the attribute mastery status of respondent *i* at time *t* and *K* is the number of attributes. For attribute 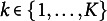 , 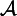 denotes the collection of attribute vectors 
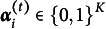
 . 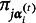 represents the probability of respondent *i* with attribute mastery status 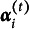 answering item *j* at time *t* correctly. The TDCM has the same components as a standard latent class model, except the structural model has an extra component: transition probabilities denoted by 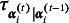 . Each 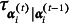 represents the probability of transitioning between different attribute mastery statuses between testing occasions for respondent *i*. The other component of the structural model, 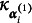 , represents the probability of having attribute status 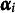 for respondent *i* at time point 1.

To evaluate intervention effects in a DCM framework, the TDCM was extended to account for multiple groups (Madison & Bradshaw, 2018b). The multiple-group TDCM is designed to assess differential growth in attribute mastery between a treatment group and a control group in a pre-test/post-test designed experiment. The general form of the multiple-group TDCM is given by 
(2)

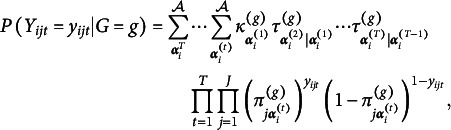

where 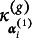 represents the probability of having attribute status 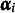 for respondent *i* from group *g* at time point *i*, 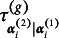 represents the probability of transitioning between different attribute mastery statuses between testing occasions for respondent *i* from group *g*, and 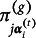 represents the probability of respondent *i* from group *g* with attribute mastery status 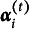 answering item *j* at time *t* correctly. The multiple-group TDCM is similar to the single-group TDCM in Equation 1, but the structural parameters are conditional on group membership *G*, which represents the potential for respondents in different groups (e.g., treatment versus control) to have different mastery transition patterns and probabilities. If the treatment group shows significantly more growth in attribute mastery than the control group, this would be evidence of a successful intervention.

While the multiple-group TDCM presents a promising methodology for evaluating intervention effects with interpretive benefits over other longitudinal psychometric modeling options, it has limitations with respect to estimation. In preliminary explorations using commercially available software (Mplus; Muthén & Muthén, 2017), estimation time and data requirements were not feasible for applications with more than two groups, more than four dichotomous attributes, more than two time points, or more complex covariate structures such as nested effects or interaction effects. This significantly limits the full utilization of the TDCM in practice as experimental designs for evaluating intervention effects often include more than two measurement occasions, more than two groups, and complex experimental designs. To provide a more flexible framework for evaluating intervention effects in a DCM framework, a modified, more flexible TDCM with a practical estimation approach is necessary.

### 2.3 Hidden Markov DCMs

For longitudinal assessment data, the hidden Markov model (HMM) posed significant advancements by accounting for dependence in attribute mastery at each time point. For instance, Wang et al. (2018) used a HMM of higher order combined with a constrained deterministic-inputs, noisy-and-gate (DINA) model to evaluate the efficacy of different learning interventions. This logistic model included covariates to account for individual differences in skill transitions. Similarly, Zhang & Chang (2020) introduced a DINA-integrated multilevel logistic HMM with random effects to account for variability in instructional methods. Transition for this model is represented by a random effects model that accounts for a student’s overall learning ability to acquire all attributes. Liu et al. (2023) proposed identifiability conditions to ensure that the DINA parameters for hidden Markov DCMs can be reliably estimated. Computationally, Yamaguchi & Martinez (2024) improved the efficiency of the hidden Markov DCM using DINA by providing a variational Bayesian inference framework. In another form of DCM, Yigit & Douglas (2021) presented a first-order HMM integrated with the generalized DINA (G-DINA) using expectation maximization to track learning trajectories, where G-DINA has provided much-desired flexibility in handling complexity such as compensatory relationships compared to the standard DINA.

Existing hidden Markov DCMs have experienced significant developments in recent years. However, strict conditions and constraints have limited their applications. Namely, the first-order Markov property for attributes only depends on the immediately preceding state and may fail to realize nuances of attribute change such as capturing long-term dependencies, accounting for non-linear learning trajectories, or other external factors (Pan et al., 2020). Although this strict assumption may not pose any issues in the scenario with two time points, the ignorance of time points other than the direct previous may not be desirable in cases with more than two time points.

In this study, we focus on the longitudinal extension of the LCDM (Henson et al., 2009), the most general and flexible model that offers a unified framework through which many previously developed DCMs, such as DINA, can be specified by placing constraints on the LCDM parameters. A hidden Markov DCM built on LCDM can be comparable to our proposed model in longitudinal assessment settings. However, a hidden Markov LCDM is restricted by the Markovian assumption for time points greater than two, as is well-known for HMMs in general (Glennie et al., 2023). Hence, our model is more flexible in interpreting the entire length of a learning trajectory (e.g., how an attribute state at the first time point relates to its third time point state) as opposed to a limited Markovian perspective of a time point and its direct preceding. In other words, a hidden Markov LCDM may lead to biased conclusions regarding respondents’ progress and transitions when the Markovian assumption is not met, whereas our model can be applied without these limitations (e.g., Pan et al., 2020).

## 3 Proposed model: extended Bayesian TDCM

We expand upon the multiple-group TDCM framework of Madison & Bradshaw (2018b) by adjusting the model formulation and offering a feasible estimation framework for model inference. Here, we utilize an updated regression-based formulation to incorporate additional covariates outside group-level treatment intervention effects.

The extended TDCM is composed of two partitions of the complete model that interact with one another: the item-attribute model and the latent transition model. For the item-attribute model, we specify LCDM to mirror the standard TDCM exactly. The proposed framework’s primary contribution to the TDCM can be found in the chosen parameterization for the latent transition model. Departing from the transition analysis for the standard TDCM shown in Equation 2, we model profile transitions in the form of discrete attribute transition types between two adjacent time points using a multinomial logistic regression for each attribute. The key appeal of the extended TDCM lies in the flexible nature of regressions to include individual-level covariates for different transition types, as demonstrated in the following empirical and simulation studies. In the following, notation is first set up in Section 3.1, then the extended TDCM formulation is described formally in Section 3.2.

### 3.1 Notation

Here, we establish the setting and notation to be used for model formulation (Table 1). We consider 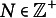 different respondents. Denote each respondent as 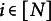 , where 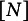 is defined as 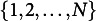 for all 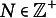 . We consider 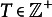 time points. Denote each time point as 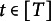 . At time point *t*, there are 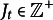 questions given to respondents. Let 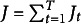 . Denote each question as 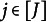 and 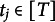 as the time when the question *j* is given. The response matrix is denoted as 
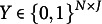
 . Denote each element as 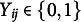 , which indicates whether the 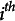 respondent answers the question *j* correctly. We consider *Y* as a random matrix and 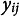 as the observed data of 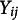 . There are 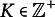 possible attributes for each question. Denote each attribute as 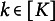 . Q-matrix 
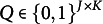
 establishes the relationship between questions and attributes, such that an element 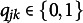 indicates whether attribute *k* is required on the question *j*. In the current article, *Q* is assumed to be known and held constant. We use 
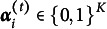
 to denote the attribute profile of respondent *i* at time *t*, and 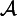 to denote the collection of all attribute vectors 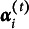 . There is a natural bijection between attribute vector 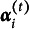 and integer classes 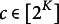 . Define vector 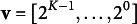 , then we can see that 
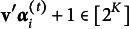
 for any 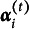 . With a slight abuse of notation, the term 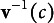 is used to denote the corresponding attribute vector associated with integer class *c*. We also use 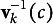 to denote the 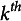 element of 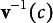 . For example, suppose 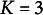 , then 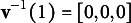 and 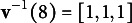 . Also, we have that 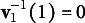 and 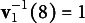 . For respondent *i* and attribute *k*, let 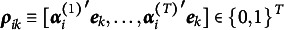 denote the respondent *i*’s latent transition vector for the attribute *k* corresponding to the set of profiles 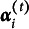 , where 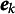 is defined as the 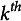 row vector of the identity matrix 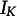 . Notice that there is a natural bijection between attribute vector 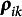 and integer classes 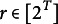 . Define vector 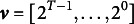 , then we can see that 
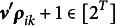
 for any 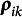 . With a slight abuse of notation, we use 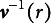 to denote the corresponding transition vector associated with integer class *r*. For example, suppose 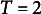 , then 
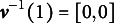
 and 
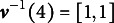
 . For each question *j*, we define a matrix 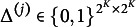 . The 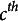 row vector of 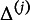 represents the design vector for attribute profile 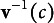 . Denote the 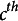 row vector of 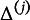 as 
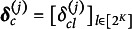
 , where 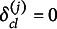 if question *j* is not testing any of all attributes in 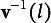 or 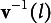 contains any attribute that 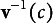 does not have. Otherwise, 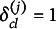 . Note that 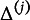 may contain columns that are 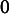 for all entries. In the following discussions, we assume there is no column of zeros in 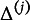 . That is, 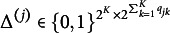 , and 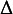 denotes the collection of all 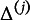 . For question *j*, we have the corresponding item parameters 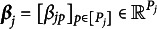 , where 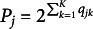 . For ease of interpretation, we assume strict monotonicity such that a latent profile class with more acquired attributes must have a response-correctness probability no less than a profile class with fewer attributes. That is, we assume 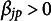 for all 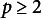 . We use multinomial logistic regressions to model latent attribute transition types. 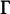 is defined as the collection of 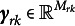 indicating the regression parameters for the 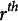 transition category of the 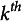 attribute, and 
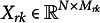
 is the design matrix for the attribute *k*. We denote the standard logistic function as 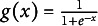 .

**Table 1:** List of notation used throughout the article

Symbol	Description
*K*	Number of latent attributes.
*N*	Number of respondents.
*T*	Number of assessments (time points).
*J*	Number of items over time.
*Y*	Item response matrix of size 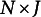 .
*Q*	Q-matrix of size 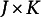 .
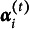	Attribute profile of respondent *i* at time *t*. 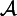 is the collection of all 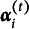 .
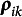	Transition pattern of attribtue *k* for respondent *i*.
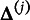	Design matrix of item *j*
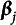	Item parameters.
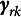	Transition parameters of transition type *r* for attribute *k*.

### 3.2 Model formulation

We define the likelihood function for the response data *Y* in the extended TDCM framework as 
(3)

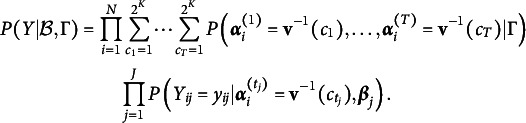



Using 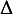 and 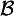 , we can define the right-side probability in Equation 3 for the correctness of a response conditioned upon the latent profile class and LCDM parameters. The logit link function models the probability to be 
(4)

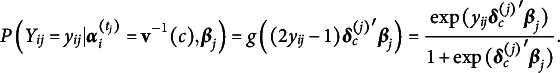



With the item-attribute portion of the model defined, the former term of the full joint probability in Equation 3 serves as the conditional probability for the transition model. Four discrete, unordered scenarios ( 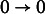 , 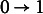 , 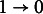 , and 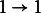 , where 0 indicates non-mastery and 1 indicates mastery and 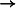 indicates transition between two time points) may occur for each binary attribute *k* from one time point to the next, thus motivating the use of multinomial logistic models. We define the conditional probability to be, 
(5)

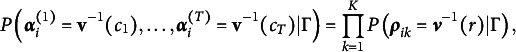

where 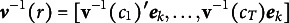 . It follows that the right-hand side of Equation 5 is modeled by multinomial logistic regressions such that 
(6)

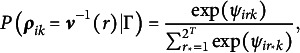

where 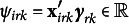 and *r* is the transition type associated with a particular attribute. The covariates in consideration pass through the design matrix 
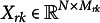
 to model the probability of 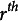 transition of attribute *k*. For identifiability purposes, the chosen default “baseline” level 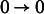 attribute transition is constrained to zero, i.e., 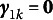 for all attributes. With this formulation, the model relaxes the Markovian assumption that hidden Markov DCMs are constrained to. The relaxation of this assumption does not present a problem for the TDCM, and would be able to account for scenarios, where an attribute status is not only dependent on its status at the previous time point. It is worth noting that the specification of 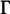 can be adjusted so that the full multinomial regression is reduced by omitting certain response levels, which can lower computational burden and improve interpretability. For instance, a study may be more interested in growth, i.e., 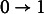 transition, and regression, i.e., 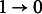 transition, than the two unchanging transition types. For time points greater than 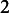 , Section 3.3 provides a more detailed discussion.

#### 3.2.1 Remark 1

The formulation of this section reintroduces 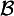 for the item-attribute LCDM, and proposes the 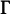 regression parameter to model latent transition using multinomial logistic regressions. It is clear from the notation that the extended TDCM is capable of relaxing time- and group- measurement invariance. LCDM parameter 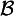 has the option to vary longitudinally, in settings, where the same test is not given at each time point. Transition probabilities modeled by 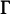 are dependent on treatment covariates and, therefore, vary across groups. While group variability is a necessity of the proposed transition model, time invariance may be applied to 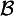 to ease interpretations. In the following section, we detail a method of simulation-based inference using an augmentation approach to logistic data.

#### 3.2.2 Remark 2

In comparison with relevant methods, the proposed TDCM extension has similar applications to the generalized hidden Markov DCM. The multilevel logistic HMM (Zhang & Chang, 2020) in particular, bears resemblance to the proposed hierarchical logistic regression-based TDCM reformulation. The distinction here is clear in covariate structure, where the HMM limits covariates to focus on learning behavior and attribute acquisition ability rather than having a flexible structure capable of group- and individual-level covariates. Moreover, the generalization that LCDM provides over DINA, often used alongside HMM combined with the Markovian assumption, are the important practical differences between the TDCM and hidden Markov DCM classes.

### 3.3 Transition types and modeling flexibility

Each additional time point in a longitudinal DCM increases the complexity of the model setup and makes interpretation more challenging. In this work, we showcase the modeling flexibility of the extended TDCM with an increasing number of assessment points. Table 2 presents the 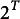 possible transition types per attribute can experience across the 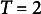 , 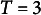 , and 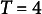 time periods.

**Table 2:** The 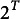 types of transitions (denoted *r*) for each attribute for (a) 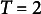 , (b) 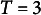 , and (c) 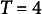

(a) 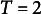				
Transition	1	2	3	4
Time 1	0	0	1	1
Time 2	0	1	0	1

*Note*: The different transitions (4, 8, and 16 types for 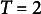 , 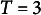 , and 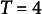 , respectively) are the possible ways that a respondent can have for a certain attribute over time.

The extended TDCM allows for flexible control of the design matrix, enabling the researcher to apply covariates selectively to specific transition types. For instance, with 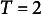 , transition type 2 ( 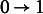 ) indicates that a student has mastered the attribute during the transition. In this case, the researcher may choose to apply student-level covariates (e.g., gender) only to this transition, while excluding covariates for the other transition types, as the primary focus is on assessing gender differences in the mastery rates of each attribute.

Transition types increase with 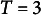 and 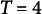 , offering additional flexibility and opportunities to explore interesting growth patterns. For example, with 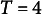 , transition type 8 ( 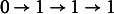 ) indicates that a student mastered and retained the attribute, which may be more interesting compared to transition 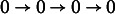 (type 1), where the student never attains the attribute, or 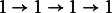 (type 16), where the student always has the attribute. The transition trajectories 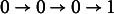 (type 2) and 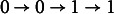 (type 4) may indicate a delayed intervention effect, where the student gains the attribute some time after the intervention. In contrast, a trajectory such as 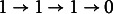 (type 15) could suggest a weak long-term effect. Another possible interpretation is to hold all time point states constant except for the first time point. Comparing the trajectories, 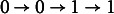 (type 4) and 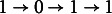 (type 12) could be helpful in determining whether having the attribute at the initial time point matters. More complex transitions, such as 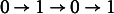 (type 6), are also possible, where a student mastered the attribute between the first and second time points, lost it between the second and third time points, and regained it between the third and fourth time points.

Note that these diverse transition patterns may not be observed with hidden Markov DCMs due to their Markovian assumptions. In contrast, the flexibility of the proposed extended TDCM allows for capturing various nuances in growth patterns and individual differences.

## 4 Bayesian estimation with Pòlya-gamma sampling

The computational complexity of the transition model’s multinomial regression formulation increases significantly for each additional attribute, and more drastically so for each additional time point. Thus, a computationally feasible estimation framework is crucial for inference. We propose a framework for Pòlya-gamma data augmentation (Polson et al., 2013) for both the item-attribute LCDM and the transition regression models, two components that compose the extended TDCM framework. The augmented data allows for Gibbs sampling to provide tractable posterior distributions for the parameters of interest, 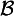 and 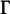 . Pòlya-gamma sampling has been applied by Jiang & Templin (2019) for the two-parameter logistic item-attribute model and by Zhang et al. (2020) for the confirmatory DINA model. Recently, Balamuta et al. (2022) presented the implementation of Pòlya-gamma data augmentation for a class of LCDM, where the *Q* matrix is inferred. To our knowledge, this work represents the first implementation of multinomial Pòlya-gamma sampling for longitudinal DCM models.

### 4.1 Pòlya-gamma sampling procedure

The Pòlya-gamma distribution is used to sample the response model auxiliary variables 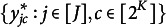 corresponding to responses for each question and attribute profile. Since there are only 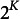 possible attribute profiles, we only need 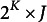 auxiliary variables instead of 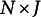 auxiliary variables. We also use Pòlya-gamma distribution to sample transition model auxiliary variables 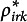 corresponding to latent transition 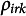 for each respondent, transition, and attribute, where we define 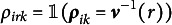.

To start, a Pòlya–gamma random variable 
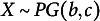
 with 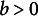 and 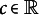 is distributed such that random variable 
(7)

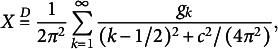

where 
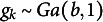
 . This distribution is key to the main result of Polson et al. (2013) that shows the Bernoulli logit link response can be augmented with the integral identity, 
(8)

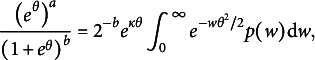

where 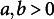 , 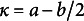 , and *w* distributed as a Pòlya–gamma random variable, i.e., 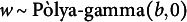 . Notice that when we have a linear function of predictors such that 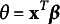 in the case of the logistic regression, the left side of Equation 8 becomes the kernel of 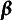 likelihood. To be exact, the contribution of a single observation *i* to 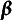 likelihood 
(9)

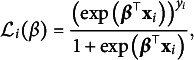

uses main Pòlya–gamma distribution property in Equation 8 to result in tractable Gaussian posterior using conjugate Gaussian prior. We adopt a Gibbs sampling algorithm in which 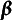 and the augmented data are sequentially sampled at each iteration, as described in Section 4.1.2. The integrand of Equation 8 may also be adapted straightforwardly to the multinomial logit link likelihood for the transition model, as seen in the following Section 4.1.4. The added efficiency of Gibbs sampling for logistic regression Bayesian inference makes Pòlya-lgamma data augmentation highly appealing. Rigorous proofs and details of the algorithm can be found in Polson et al. (2013).

In the extended TDCM case, computations of posterior distributions for the parameters in 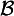 and 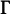 loosely follow the Pòlya-gamma framework of Balamuta et al. (2022). Consistent with that framework, the priors of the model are defined to be 
(10)

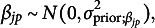



(11)

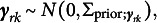

where we choose 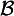 priors 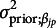 for each 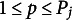 and 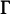 priors 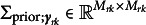 . In accordance with the monotonicity condition for 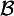 , elements of 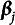 are updated sequentially. Monotonicity here is based on an “intuitive plausible assumption” (Rupp et al., 2010), and allows the modeler to decide whether to impose the constraint based on its appropriateness in context or even additional efforts required for model calibration. To be consistent with the motivating setting, the posterior distributions we use are truncated such that 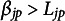 at each iteration, where 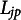 is set such the full conditional distribution for intercept and interaction terms are unrestricted with only main effects restricted (Balamuta et al., 2022). These exact posteriors distributions are defined in Section 4.1.2.

The MCMC procedure for the extended TDCM is hierarchical, reflecting the hierarchy of the response model, which depends on the transition models in Section 3.2. The main Gibbs sampling procedure for the response LCDM samples 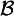 and 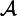 , while the transition parameter 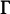 is sampled in the second-level procedures within the main MCMC for each of the *K* transition regressions using the same Pòlya-gamma scheme. To decrease the drastic computational cost of an additional attribute, the *K* second-level procedures are set to have drastically fewer iterations (*m*) compared to the LCDM Gibbs sampling iterations (*M*) such that 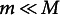 . As long as *M* is large enough to account for burn-in, our efforts show that posterior samples achieve convergence for small *m*. The minimum iteration 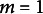 is used in all following empirical and simulation settings to reach convergence. However, *m* may be increased to a greater value (e.g., 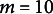 ) with increased complexity to transition parameter 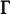 .

The following procedure samples three sets of parameters defined in the previous section: 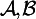 , and 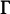 . The first step to sample 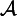 is dependent on both the LCDM and transition regressions, while the augmentation scheme to sample 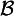 and 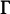 depends on the sampled 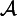 . Since each step relies on conditional distributions given the other parameters, each variable within 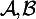 , and 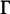 should be randomly initialized in advance. The procedure is fully implemented using R and can be accessed through GitHub at [anonymized]. A single full iteration of the Gibbs sampler for 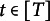 is described in the three steps below. Additional details are provided in Appendix A on the derivation of the posterior distribution for the proposed MCMC procedure. A summary of wall-clock runtimes for all empirical and simulation models is provided in Table 10 in the Supplementary Material, illustrating the practical efficiency of the proposed sampling procedure.

#### Step 1: sample 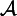


4.1.1

Each of the *M* iterations begins with sampling latent attribute profiles 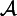 . For each *i* and *t*, 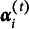 is sampled from the conditional distribution 
(12)

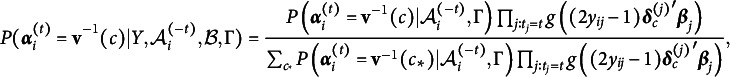

where 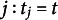 being question from time *t*. We define the set of profiles of respondent *i* excluding time *t* as 
(13)

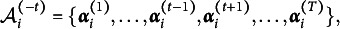



Further, the conditional probabilities can be calculated with the implicit attribute transition probabilities from one time point to its next using transition regression parameter 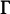 . Equation (12) is computed such that 
(14)

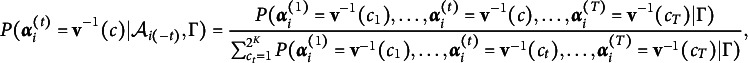

which involves the calculation of the joint probabilities 
(15)

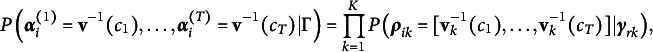

where 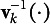 is defined in Section 3.1. The probabilities in the product are calculated according to the transition model defined in Equation 6. The resulting 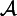 carries attribute profiles for respondents at each of the *T* time points.

#### Step 2: sample 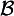


4.1.2

Following 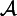 , we sample item-attribute LCDM parameters 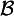 by first sampling Pòlya–gamma auxiliary response variables 
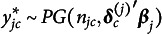
 , where 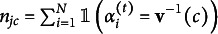 for each question *j* and profile *c*. Then, 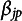 can be sampled from the truncated normal posterior distribution 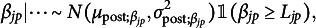 where 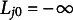 and is set to zero for all other *p* for monotonicity constraint. The posterior has the derived parameters 
(16)

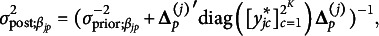



(17)

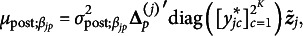

where 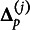 is the 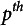 column vector of 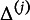 , namely, 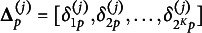 . 
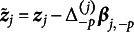
 with 
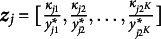
 and 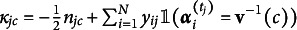. Here, we denote 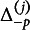 is 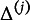 with the 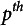 column removed and 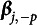 is 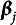 with the 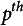 entry removed.

Note the sampling of 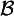 here is the general case, where we specify a distinct set of LCDM parameters for each time point *t*, thus allowing tests at different time points to have different *Q*. In the more common setting, where *Q* remains the same over time, the following passage provides the posterior distributions such that 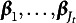 encompasses model parameters over all time points. The unconstrained version of the proposed model is comparable to that of the standard TDCM, and the time-constrained adjustment to sampling 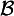 is straightforward to implement as well.

#### Time-constrained 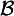


4.1.3

The general case for sampling 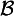 in Section 4.1.2 allows for the flexibility of varying *Q* matrices over time, i.e., a unique set of questions at each time point. However, the motivating setting of Madison & Bradshaw (2018b) focuses on assuming a constant *Q* matrix for all time points, thus fixing the 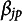 constant over time. We show here that the time-varying 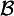 may be straightforwardly altered to account for that. Let us consider the alternate case, where the *J* questions are repeated over time. In this case, we have 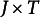 questions in total. With a slight abuse of notation, we use 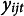 to denote the correctness of respondent *i*’s response to question *j* at time *t*. Then, 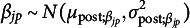 for 
(18)

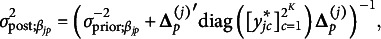

where 
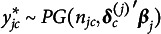
 with 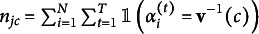. 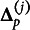 is defined in the same way as in the previous setting. 
(19)

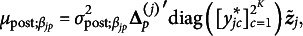

where 
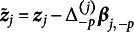
 with 
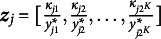
 and 

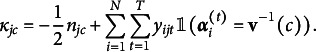



The posterior distribution here is directly adapted to Step 2 of the above procedure, with the remaining steps held constant. Implementations of this article default to constraining the extended TDCM by fixing 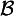 , while time-varying 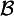 is an option that can be easily called upon as well.

#### Step 3: sample 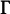


4.1.4

In the final step, 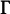 parameters for transition multinomial logistic regressions are sampled. This is done by first sampling the Pòlya-gamma auxiliary variables 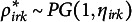 where 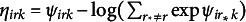 , and recalling that 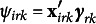 . Also recall that we constrain 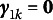 for all *k* to serve as the “baseline” transition. It follows that 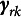 can be sampled from the posterior distribution 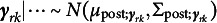 with parameters 
(20)

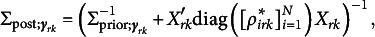



(21)

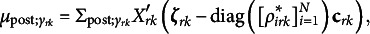

where 
(22)

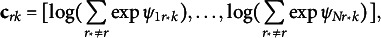

and 
(23)

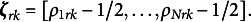



We note here that the step to sample 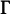 is repeated for each of the *K* regression models, and a single taken sample is embedded within each *M* Gibbs iteration. The three MCMC inference steps sampling each latent attribute 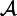 , item-attribute 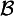 , and transition regression 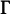 are iterated *M* times to produce full posterior samples.

While the number of Gibbs sampling iterations required is case-specific, the efficiency that Pòlya-gamma provides flexibility in increasing iterations without a costly sacrifice to computation. In each of the following empirical and simulation studies, 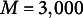 with 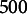 burn-in iterations is used and often proved to be more than enough for the model to reach convergence. Convergence is assured in each of the following scenarios through careful checking of trace plots.

## 5 Empirical validation

For empirical validation, we first apply the extended TDCM to the two cases examined in Madison & Bradshaw (2018a) and Madison & Bradshaw (2018b), with the single-group and multiple-group settings, respectively. In both cases, the results presented here are validated by comparing them with the previously reported findings, assuming the prior results are considered valid. We showcase the additional insights provided by the proposed reformulation of the TDCM, which is primarily reflected in the transition regression parameter 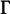 . Further, we use a separate data set with the same assessment to demonstrate the extended TDCM’s main contribution: its flexibility in including covariates in a multiple-group setting. Across all three scenarios, we assess model fit using posterior predictive checks, which represent an additional advantage of the proposed approach over the existing TDCM framework.

The main goal of the empirical section is to ensure that the proposed model provides the exact results that the standard TDCM was able to, while opening a window to the more complex simulation scenarios that emphasize the extended TDCM’s added contributions. As we find in the following, the empirical data used here have no or a simple covariate structure under only two time points. The extended TDCM produces satisfactory results and demonstrates its suitability for the more complex settings that follow.

### 5.1 Single-group setting

#### 5.1.1 Data and analysis

We use the mathematics assessment data from the studies of Bottge et al. (2014) and Bottge et al. (2015). The studies aim to evaluate the effectiveness of an instructional method (enhanced anchored instruction [EAI]) on mathematics exams over the course of 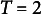 time points one year apart. The total number of students is 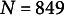 , of which 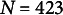 received EAI and act as the treatment group while the control group of 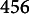 students did not receive the instructional treatment. The mastery of four ( 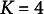 ) attributes of interest is implicitly measured: RPR, MD, NF, and GG. Each with 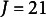 questions, the two tests measuring attributes are defined by the same *Q* matrix matching items to attributes, shown in Table 3. We see here that none of the items measure more than one attribute, meaning the item-attribute regression parameter 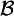 contains only main effects and no interaction terms.

**Table 3:** *Q* matrix for empirical data (Bottge et al., 2014, 2015) indicating attribute requirements for each 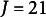 test item questions

Item No	1	2	3	4	5	6	7	8	9	10	11	12	13	14	15	16	17	18	19	20	21
RPR	1	0	0	0	0	0	0	0	0	0	0	0	1	1	0	0	1	0	0	0	0
MD	0	1	1	0	0	0	0	0	1	1	1	1	0	0	0	0	0	0	0	0	0
NF	0	0	0	1	1	1	1	1	0	0	0	0	0	0	0	0	0	0	0	0	0
GG	0	0	0	0	0	0	0	0	0	0	0	0	0	0	1	1	0	1	1	1	1

*Note*: The four attributes indicated are: ratios and proportional relationships (RPR), measurement and data (MD), number systems (NF), and geometry and graphing (GG).

The same data set and *Q* matrix were used for the single-group TDCM in Madison & Bradshaw (2018a). In the single-group setting, where no group membership is considered, the effect of the educational EAI treatment is not of interest and thus the transition regressions are simplified as explained in the following 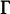 posteriors. The Gibbs sampling procedure for the extended TDCM defined in Section 4 is followed here. We use 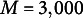 as the number of iterations for the MCMC procedure, with the first 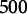 burn-in samples removed from posterior estimates. The number of iterations appears adequate, given the speedy convergence of Gibbs samplings as seen in trace plots in the Supplementary Material. For Bayesian inference, the standard deviations of the normal distribution priors on 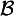 and 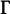 are 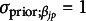 and 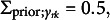 respectively, such that the priors are approximately non-informative with reasonable constraint.

#### 5.1.2 Replication

**Figure 1:**
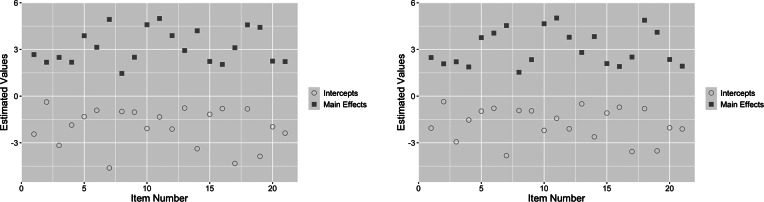
*Comparison of* 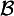 *point estimates between the standard TDCM(left) and the extended TDCM (right) for the single-group setting.*

We replicate key results shown by the original study of Madison & Bradshaw (2018a) and present additional results demonstrating extended TDCM’s added value. The comparison begins with point estimates for 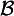 in Figure 1. The standard TDCM results on the left were obtained using the Mplus code from Madison & Bradshaw (2018a) (these results are identical to those presented in Figure 2 of Madison & Bradshaw (2018a)). The extended TDCM point estimates are the averages of the 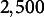 burned-in posterior samples outputted by the Pòlya-Gamma algorithm. The results here are approximately identical for both the intercepts and main effects for each of the 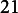 questions.

**Figure 2:**
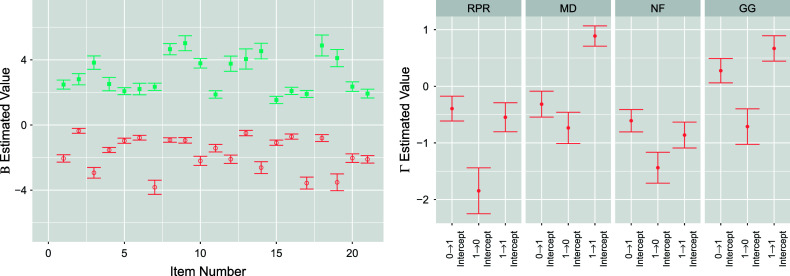
*Posterior sample distributions for* 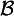 *(left) and* 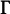 *(right) bounded by two standard deviations.* *Note*: For 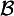 , each question item contains an intercept in red and a main effect in blue. The four segments for 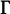 plot correspond to the four attributes: RPR, MD, NF, and GG. The indices within each attribute segment denote transition 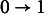 intercept, 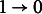 intercept, and 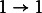 intercept, respectively.

#### 5.1.3 Further flexibility

While this is encouraging, we further show the appeal of extended TDCM by noting that the proposed model offers an additional set of regression parameters 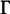 for transition, on top of LCDM parameters 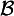 . For both sets of parameters, full posterior samples are available and show convergence evidenced in the Supplementary Material. Point estimates and their 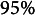 credible intervals are shown for both sets of parameters in Figure 2 for the burned-in samples. We see that the variability of parameters remains roughly constant among all parameters, with 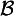 variability increasing slightly as point estimates depart from zero.

The full posterior distributions of 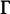 , shown on the right side of Figure 2, serve as a main appeal of our TDCM extension. For the single-group case, we note that the simplified transition regressions can be considered null models in that the multinomial regression for each attribute contains only the intercept. The figure shows four segments denoted by the labels on the top showing 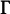 for each of the 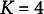 attributes. Within each segment, the indices on the bottom denote the intercepts for the transition 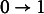 , 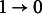 , and 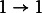 , respectively, with the 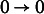 transition being the baseline level and 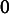 indicates non-mastery, 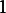 indicates mastery, and 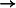 indicates transition between two time points. The converged estimates here show approximate constant variance. It is also seen that the attribute regress transition 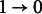 , the loss of an attribute between the two time points, is lower in log-odds than other transition types. This makes sense in context given the underlying behavior that some respondents received treatment that helped them answer the questions between the two time points. These estimates are greatly useful in the context of regression, and they further lead to direct conversion into transition probabilities conditioned on whether an attribute is acquired at the initial time point.

The conditional transition probability is a direct function of 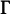 posteriors by nature of the logistic link. In the original study (Madison & Bradshaw, 2018a) where latent transition were not modeled by regressions, the transition probabilities were attained by comparing latent profiles 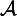 between the two time points. These are provided on the left side of Table 4, with the probability of transitioning conditioned on the presence of attributes at the initial, pre-test time point. In comparison, the transitioning probabilities posterior distributions derived from 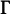 posteriors are presented on the right side of Table 4, with point estimates and attached standard deviations in parentheses. These posteriors here are not naturally conditional; therefore, conditioning by force leads to the same standard deviation for estimates that share common attribute status at the initial time point (e.g., standard deviation given no attribute 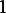 are both 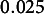 in Table 4). Precisely, in comparison, the extended TDCM provides the advantage of full posteriors for transition probabilities, whereas the standard TDCM only produces point estimates.

**Table 4:**
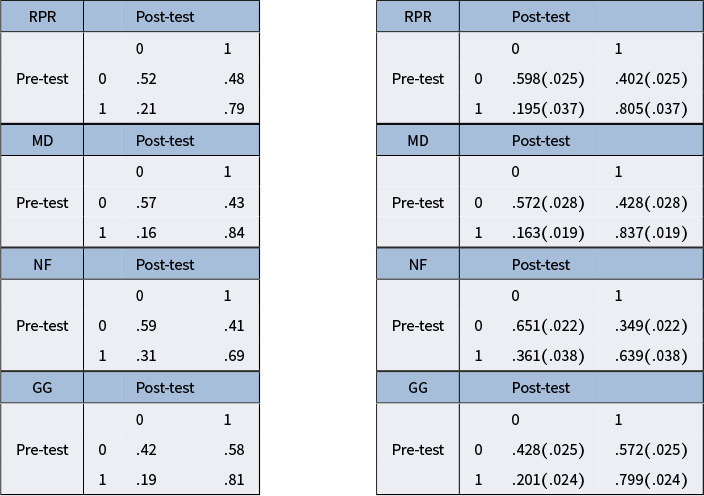
Comparison of implicit conditional transition probabilities for each 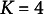 attributes of the single-group empirical study for the standard TDCM (left) and extended TDCM (right) posterior means and standard deviations in parenthesis

*Note*: The matrices are labeled with 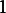 being attribute mastery and 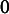 otherwise.

#### 5.1.4 Goodness-of-fit

**Figure 3:**
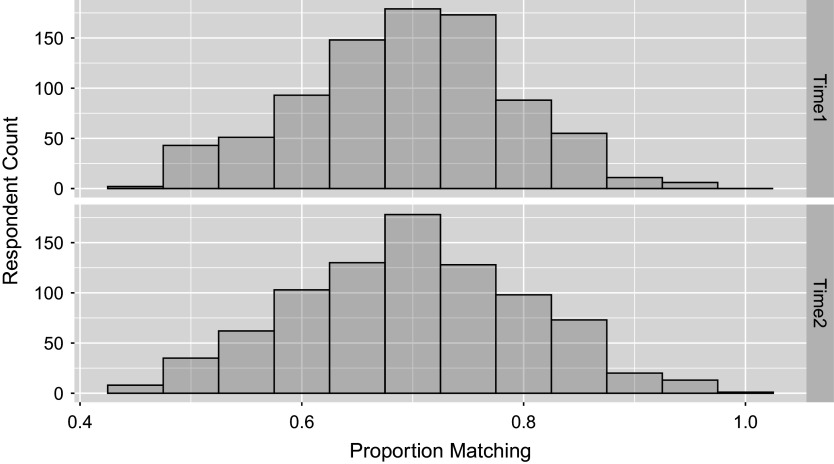
*Distribution of probability of extended TDCM fit to match the empirical response data average for* 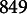 *respondents averaged over* 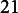 *total questions, done for both time points of the study in the single-group setting.*

**Figure 4:**
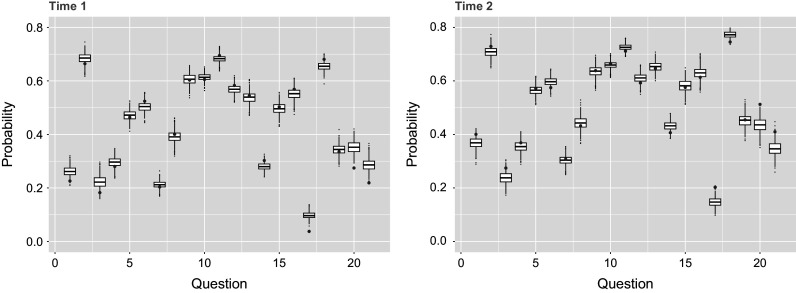
*Percentage of* 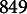 *respondents that answered each of the* 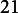 *questions correctly, with the value for the original data shown by the red points and simulated posterior predictive data shown by distributions.* *Note*: Results for both time points are shown for the single-group setting.

The Bayesian estimation approach adopted here enables the evaluation of model fit through posterior predictive checks, offering an additional advantage over the standard TDCM framework. The use of posterior predictive checks in this empirical study is loosely grounded in the framework proposed by Gelman et al. (1996). In psychometric applications, such checks have general precedent in educational measurement settings (Sinharay et al., 2006) and have been more specifically applied within the context of CDMs (Park et al., 2015). Although there are no treatments or covariates for the single-group case, null models themselves can be used to perform this predictive check. Here, the posterior predictive LCDM data are simulated and compared with the actual item-attribute data. We take the final 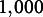 post-burn-in posterior samples and simulate posterior predictive data for each iteration. The one-to-one match provides percentage matching between the real data and 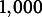 sets of posterior predictive data, which can be summarized in that the posterior predictive data matches on average 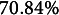 of actual data in the first time point, and on average 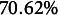 of actual data in second time point. The matching probabilities can further be broken down by question in Table 5 and by person in Figure 3. Alternative to percentage matching and done similarly in Sinharay et al (2006, Figure 6), Figure 4 uses the 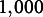 posterior predictive data sets to observe the percentage of respondents who answer correctly for each question. These posterior predictive distributions align reasonably well with the observed data, though they reflect variation due to simulation noise. However, the percentage matching at around 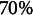 also indicates room for improvement. Overall, the moderate match rate of approximately 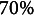 suggests that the model captures key response patterns, though improvements may be possible by incorporating individual-level covariates, which are not available in the present dataset.

**Table 5:** Probabilities of extended TDCM fit to match the empirical response data are averaged over all 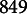 respondents for each of the 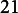 test items, done for both time points of the study in the single-group setting

Item No	1	2	3	4	5	6	7	8	9	10	11	12	13	14	15	16	17	18	19	20	21
Time 1	.71	.64	.71	.65	.69	.67	.84	.57	.63	.84	.85	.77	.62	.81	.61	.59	.88	.77	.74	.64	.66
Time 2	.66	.69	.66	.62	.73	.73	.79	.58	.67	.86	.88	.76	.69	.76	.62	.63	.75	.85	.67	.62	.59

*Note*: Posteriors for latent attribute 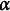 are used to identify profiles at each time point for the respondents, after which 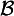 posteriors infer logistic fits to be compared with the empirical data of interest.

To further evaluate the predictive performance of the extended TDCM beyond posterior predictive checks, we report the area under the curve (AUC) and Brier Score at each time point. These predictive checks are commonly used by CDMs, such as Liang et al. (2023) using both in combination. In our analysis, the AUC across 21 test items was 0.868–0.87 (Time 1) and 0.877–0.886 (Time 2). The average Brier scores were 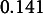 for Time 1 and 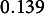 for Time 2. Taken together with the posterior predictive checks reported above, these results provide converging evidence for the model’s predictive adequacy across both time points.

### 5.2 Multiple-group setting

#### 5.2.1 Data and analysis

We now consider a multiple-group setting, where the key results are compared against those from the standard multiple-group TDCM (Madison & Bradshaw, 2018b). The same mathematical education data set is considered, and now with the educational intervention considered between the two time points. That is, we consider the group-level treatment covariate such that 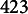 respondents receive the EAI intervention (treatment group), while the remaining 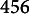 respondents receive no intervention (control group). By treating the mathematical education intervention as a covariate, it can be applied to each of the 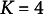 transition regressions. It follows that we have the option to apply the covariate for select transition types, intuitively the changing transition 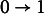 and 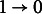 , while ignoring the covariate for unchanging transition levels 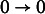 (baseline), and 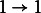 . This is done in our case for computational simplicity, as seen further in detail from the structure of 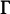 . Priors remain the same as those used by the single-group case, with 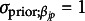 and 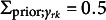 . The same number of 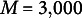 Gibbs samples were computed, with the first 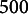 removed as burn-in samples. Trace plots for random indices of 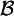 and 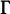 are provided in the Supplementary Material showing proper convergence.

#### 5.2.2 Replication

**Figure 5:**
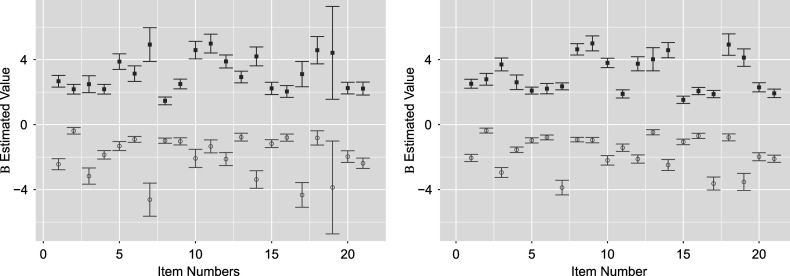
*Posterior* 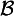 *distributions plotted using the TDCM results (obtained using the Mplus code from Madison & Bradshaw (2018b)) bounded by two reported standard errors (left) is compared to the extended TDCM bounded by two posterior standard deviations (right).* *Note*: Red indicates the question intercepts, while blue indicates the main effects.

The resulting estimates for 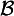 are shown in Figure 5 for both the standard multiple-group TDCM (left) and its extension (right). The results on the left for the standard multiple-group TDCM were obtained using the Mplus code from Madison & Bradshaw (2018b) (which are identical to the graphical representation of Madison & Bradshaw (2018b, Table 2)). Note that the results of the extended TDCM shown here are nearly identical to the extended TDCM 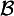 posteriors for the single-group data set in Figure 2 in the previous section.

We note, however, that the same is not true between the standard single-group and multiple-group TDCM, with their 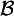 estimates being noticeably different from one another. This is seen by comparing Figure 5 (left) and Figure 2 (left). Intuitively, these results favor the extended TDCM over the standard multiple-group TDCM since the addition of a covariate on the same data set should not affect the attribute-response relationship reflected by 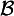 . More so, we see in the standard TDCM results that items 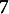 and item 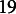 show particularly large variability in both 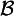 intercept and main effect. The extended TDCM does not reflect this in contrast.

#### 5.2.3 Further flexibility

**Figure 6:**
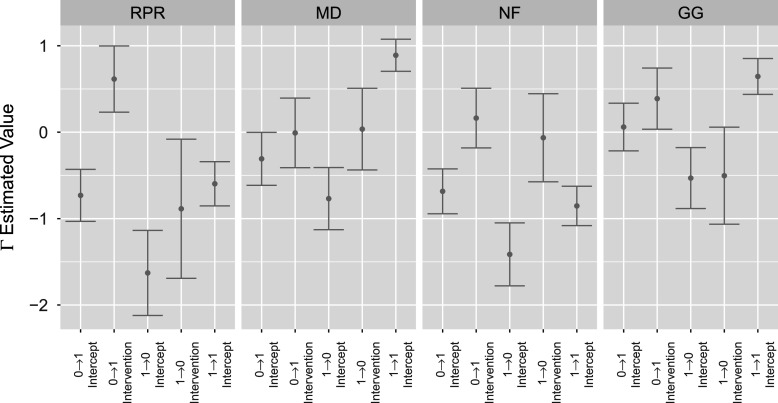
*The four segments for* 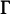 *plot correspond to the four attributes: RPR, MD, NF, and GG.* *Note*: The indices within each attribute segment denote transition 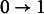 intercept, 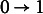 intervention, 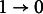 intercept, 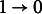 intervention, and 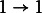 intercept, respectively.

The complementing set of attribute transition 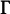 posteriors for the extended TDCM are shown in Figure 6. In the same manner as single-group 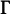 in Figure 2 (right), the multiple-group 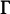 is split into quadrants for each attribute. The indices explicitly show the adaptation of the treatment covariate, where transitions 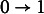 and 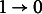 receive the covariate. As is the goal for the EAI treatment, the treatment effects clearly indicate that the treatment increases the log-odd for the attribute gain transition 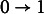 and decreases the log-odd for attribute loss transition 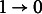 for attributes RPR, NF, and GG. The treatment has near-zero effects for attribute NF, and it follows that its intercepts are approximately those of the single-group in Figure 2 (right). These 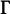 posteriors can be further compared using attribute transition probabilities.

To demonstrate the attribute transition aspect of the treatment effect, the authors of the standard TDCM show the difference in transition probability between the treatment and control groups. Attribute probabilities for non-mastery to mastery (transition 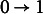 ) and mastery to non-mastery (transition 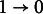 ) are displayed for each attribute in Madison & Bradshaw (2018b, Table 5). The 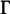 posteriors are used again for the extended TDCM to produce transition probabilities, just as done for single-group previously. The two sets of probabilities for the control and treatment groups are differentiated using two sets of design matrices for 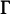 for whether treatment is received. Table 6 shows the full comparison in these transition probabilities between the proposed extension and its standard form. The table shows that the results are similar, which is encouraging for the extended TDCM since the standard TDCM derives transition probabilities using sampled attribute posteriors rather than transition regression posteriors.

**Table 6:** Transition probabilities for 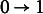 and 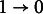 transitions between the control and treatment groups, compared between the standard multiple-group TDCM (Standard) and the extended TDCM (Extended)

		Control		Treatment	
Transition	Attribute	Standard	Extended	Standard	Extended
	RPR	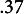	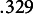	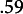	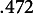
Non-mastery to	MD	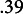	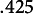	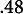	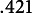
Mastery 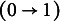	NSF	.38	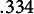	.45	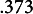
	GG	.48	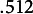	.69	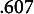
	RPR	.27	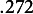	.14	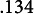
Mastery to	MD	.17	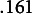	.15	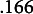
Non-mastery 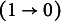	NSF	.34	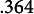	.28	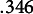
	GG	.24	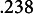	.15	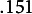

#### 5.2.4 Goodness-of-fit

**Figure 7:**
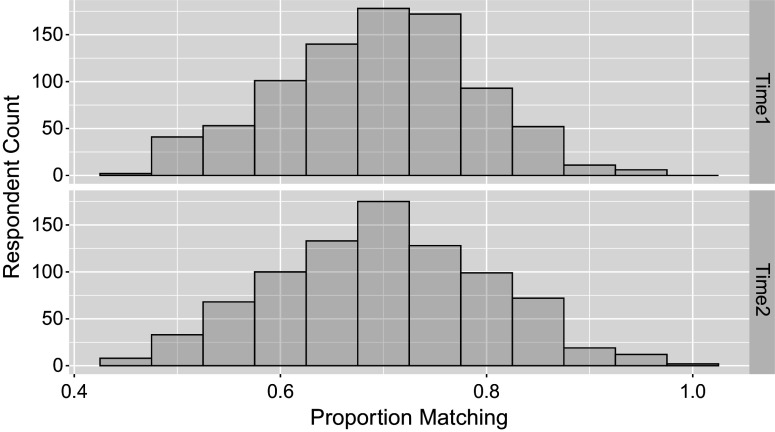
*Distribution of probability of extended TDCM fit to match the empirical response data average for* 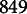 *respondents averaged over* 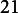 *total questions, done for both time points of the study in the multiple-group setting.*

**Figure 8:**
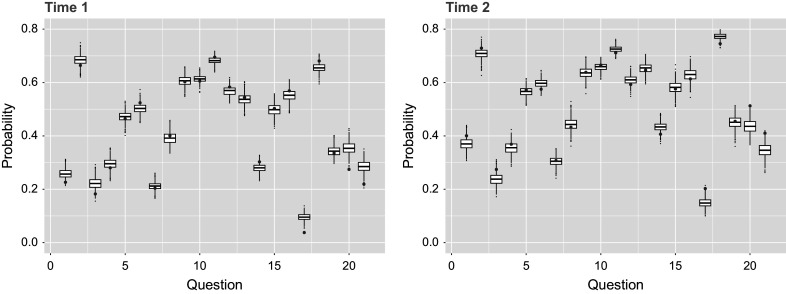
*Percentage of* 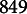 *respondents that answered each of the* 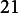 *questions correctly, with the value for the original data shown by the red points and simulated posterior predictive data shown by distributions.* *Note*: Results for both time points are shown for the multiple-group setting.

The predictive check done for the previous single-group example is applied for the multiple-group model case, by questions in Table 7 and by respondents in Figure 7. Here, we see that on average 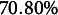 of predictive data matches at the first time point, and 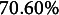 at the second time point. Looking at the percentage answered correctly by respondents in Figure 8, the posterior predictive data results match approximately the original data results just as seen in Figure 4 for the single-group case. Furthermore, the AUC across 21 test items was 0.867–0.878 (Time 1) and 0.877–0.887% (Time 2), while the average Brier scores were 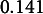 (Time 1) and 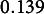 (Time 2). We see that AUC and Brier score match closely with those of the single-group case as well. Predictive evidence here indicates a moderately strong fit of the extended TDCM with multiple groups to the data.

**Table 7:** Probabilities of extended TDCM fit to match the empirical response data are averaged over all 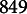 respondents for each of the 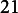 test items, done for both time points of the study in the multiple-group setting

Item No	1	2	3	4	5	6	7	8	9	10	11	12	13	14	15	16	17	18	19	20	21
Time 1	.72	.64	.71	.65	.69	.67	.84	.57	.64	.84	.85	.77	.62	.80	.60	.59	.88	.77	.74	.64	.66
Time 2	.67	.69	.66	.63	.73	.73	.79	.58	.67	.85	.88	.76	.69	.76	.62	.62	.75	.85	.68	.61	.59

*Note*: Posteriors for latent attribute 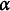 are used to identify profiles at each time point for the respondents, after which 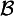 posteriors infer logistic fits to be compared with the empirical data of interest.

### 5.3 Multiple-group with covariates setting

#### 5.3.1 Data and analysis

Next, we consider a separate dataset from the same studies (Bottge et al., 2014, 2015), which includes two student-level covariates in a multiple-group setting. Incorporating additional student-level covariates, introduces extra complexity into the transition parameter 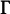 structure for the extended TDCM. Given the same mathematics test, i.e., the same *Q*-matrix for attributes RPR, MD, NF, and GG, as those used in the single-group and multiple-group settings, this unique set of response data was taken and accompanied by the student-level covariates gender (male and female) and binary membership status in the English as a Second Language (ESL) program. Observations are taken for 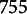 students in this data set, of which 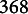 students receive the EAI treatment and 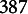 do not.

The EAI intervention treatment of interest is treated as the same level as the student-level covariates in the extended TDCM, thus the focus of this setting lies in the additional dimensions of the 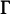 structure. Intervention treatment and the two covariates are enforced onto the 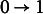 and 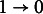 transitions, as is consistent with the previous empirical examples. In that same respect, 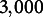 Gibbs sampler iterations were taken with the first 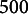 reported as burn-in. Priors used are 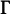 are 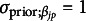 and 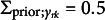 .

#### 5.3.2 Results

**Figure 9:**
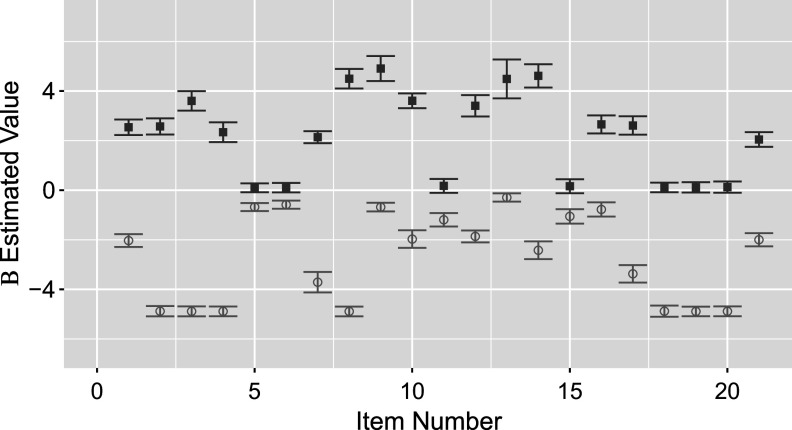
*Posterior* 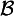 *distributions of extended TDCM bounded by two posterior standard deviations (right).* *Note*: Red indicates the question intercepts, while blue indicates the main effects.

**Figure 10:**
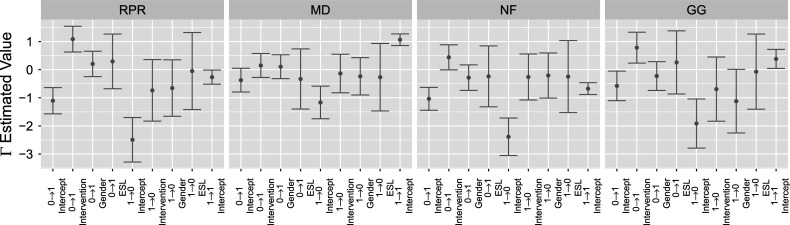
*The four segments for* 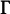 *plot correspond to the four attributes: RPR, MD, NF, and GG.* *Note*: The indices within each attribute segment denote transition 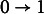 intercept, 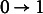 intervention, 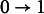 gender, 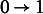 ESL, 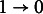 intercept, 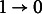 intervention, 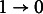 gender, 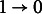 ESL, and 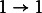 intercept, respectively.

Figure 9 shows the posterior distribution of 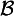 parameters. Of more interest is Figure 10, showing the regression log-odds effects of intervention as well as the covariate effects of gender and ESL. The EAI intervention is seen to boost the log-odds of a 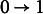 transition for all attributes except MD, for which its effect does not significantly depart from zero. It is also reasonable here that the intervention does not contribute to losing an attribute, i.e., the 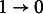 transition. For student-level covariates, gender does not seem to be an influential factor for attribute transition. However, being in an ESL program does seem to inhibit students from gaining an attribute (i.e., 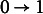 transition). This is reasonably so as the English ability would factor into how well a student learns in an English environment. It is also worth noting that if a student already has an attribute, being in an ESL program does not cause them to lose the attribute, as seen by the 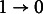 ESL effects for each attribute in Figure 10.

#### 5.3.3 Goodness-of-fit

**Figure 11:**
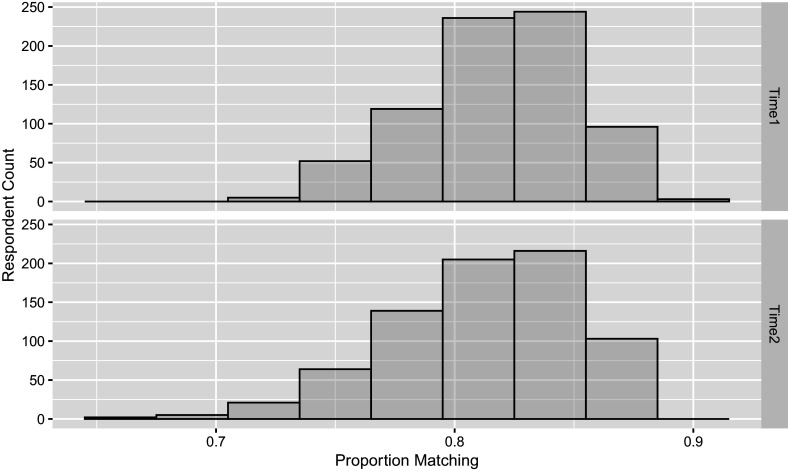
*Distribution of probability of extended TDCM fit to match the empirical response data average for* 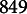 *respondents averaged over* 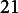 *total questions, done for both time points of the study in the multiple-group covariates setting.*

**Figure 12:**
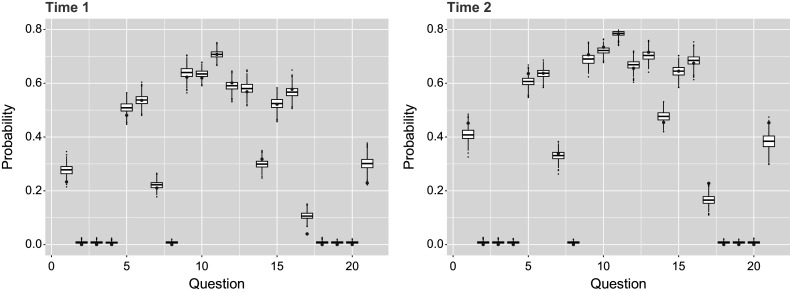
*Percentage of* 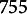 *respondents that answered each of the* 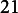 *questions correctly, with the value for the original data shown by the red points and posterior predictive data shown by distributions.* *Note*: Results for both time points are shown for the multiple-group with covariates setting.

The posterior predictive checks are based on response data generated using the original design matrix and posterior draws from the extended TDCM, consistent with the approach used in the two earlier empirical examples. The result here states that the posterior predictive data matches on average 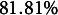 of the actual data at the first time point, and 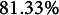 at the second time point. Table 8 provides detailed matching percentages by question, which is reflected by the histograms composed of matching percentages for individual students in Figure 11. The true probabilities of questions answered correctly in Figure 12 show the improvement from time point 1 to time point 2 being captured by the burned-in posterior predictive data sets. In addition, we observe AUC of 0.947–0.953 (Time 1) and 0.947–0.953 (Time 2) with Brier scores of 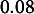 and 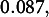 respectively, showing reliable posterior fits.

**Table 8:** Probabilities of extended TDCM fit to match the empirical response data are averaged over all 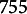 respondents for each of the 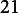 test items, done for both time points of the study in the multiple-group covariates setting

Item No	1	2	3	4	5	6	7	8	9	10	11	12	13	14	15	16	17	18	19	20	21
Time 1	.72	.99	.99	.99	.66	.67	.85	.99	.64	.83	.85	.76	.62	.79	.67	.67	.87	.99	.99	.99	.67
Time 2	.64	.99	.99	.99	.70	.73	.80	.99	.67	.85	.88	.75	.69	.73	.68	.70	.72	.99	.99	.99	.59

*Note*: Posteriors for latent attribute 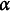 are used to identify profiles at each time point for the respondents, after which 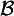 posteriors infer logistic fits to be compared with the empirical data of interest.

To parallel the comparison of posterior predictive checks for the single-group and multiple-group models, we additionally fit a simplified model without covariates to the data analyzed in this section. That is, we ignore the intervention effect along with student-level covariates for gender and ESL participation from the transition 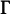 structure. The percentage matching by question produced by this model’s posterior predictive data using the final 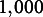 post-burn-in samples is shown in Table 9. Compared to full model results in Table 8, we see almost no difference in the posterior fitting (including AUC and Brier score) here. This lack of difference is also seen for the single-group and multiple-group cases when comparing Tables 5 and 7. This may be caused by moderate 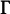 values associated with the Bernoulli intervention effect and student-level covariates of our empirical data.

**Table 9:** Probabilities of extended TDCM fit to match the empirical response data are averaged over all 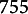 respondents for each of the 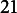 test items, done for both time points of the study in the single-group, no-covariate setting

Item No	1	2	3	4	5	6	7	8	9	10	11	12	13	14	15	16	17	18	19	20	21
Time 1	.72	.99	.99	.99	.66	.67	.85	.99	.64	.83	.85	.76	.62	.79	.67	.67	.87	.99	.99	.99	.67
Time 2	.64	.99	.99	.99	.70	.73	.80	.99	.67	.85	.88	.75	.69	.73	.68	.70	.72	.99	.99	.99	.59

*Note*: Posteriors for latent attribute 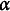 are used to identify profiles at each time point for the respondents, after which 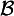 posteriors infer logistic fits to be compared with the empirical data of interest.

This model fits the data better than the simpler single-group and multiple-group models discussed earlier, as seen in the goodness-of-fit analysis. However, a direct comparison between this and the other two cases is not ideal, as different datasets were used. Additionally, the goodness-of-fit assessment was conducted for each case to demonstrate the capabilities of the proposed extended Bayesian TDCM framework. The focus of the evaluation is not on the actual fit of the models, as 1) these are existing models, and 2) the goal is to promote the extended, flexible modeling framework, rather than the models themselves.

## 6 Simulation study

In the simulation studies for the extended Bayesian TDCM, four simulation settings are used to evaluate the performance of the proposed extended TDCM. These scenarios demonstrate the potential for TDCM to model data with additional complexity compared to the empirical data analyzed in Section 5. In each setting, we provide the posteriors for 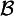 and 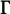 as is done in the previous section. With true parameter values that simulated data used available to us, we draw focus on parameter recovery rates, or credible interval coverage probability, shown for each setting. In the favorable scenario, the extended TDCM would be able to produce posterior samples with credible intervals having high coverage of true simulation parameters.

The simulation settings are organized as follows: We begin with a non-complex two time point scenario that includes no item-attribute interaction terms with only an intervention treatment, mirroring the previous empirical set-up (Setting 1). The model then becomes more flexible and increases the number of parameters by introducing item-attribute interaction effects (Setting 2). This is followed by the inclusion of additional covariates as is the appeal of logistic regressions for attribute transition (Setting 3). We further push the extended TDCM to account for data with three time points, which drastically increases the number of transition regression parameters (Setting 4).

In each simulation setting, data is generated using 800 respondents ( 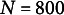 ), 21 questions ( 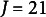 ), and 3 attributes ( 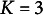 ). A total of 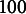 data sets, each of which includes item-correct response data *Y*, and respondent profiles 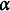 , are simulated from fixed 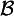 item-attribute parameters and 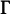 transition regression parameters. We set prior for each parameter with intention to be approximately non-informative, such that 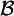 and 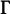 have the respective standard deviation priors 
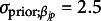
 and 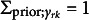 for all four settings. Each set of data *Y* and attribute profiles 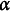 exhausts 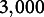 Gibbs sampling iterations with 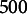 burn-in samples. The remaining 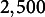 posterior samples are taken in mean, and the distribution of 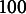 simulation means are reported. These distributions aim to estimate the true values of 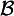 and 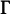 , thus the coverage rate of each simulation’s accounting of the true parameter values is reported as well.

The results following indicate high credible interval coverage by posteriors produced in all simulation cases, even for three time points. This indicates the extended TDCM and its inference framework to be computationally proficient. In addition to model fit, we report total computation times for all simulation settings in Table 10 in the Supplementary Material. In one comparison, the extended TDCM for single-group (9 min 52 sec) requires less than half of the standard TDCM (20 min 54 sec). Further results show that the proposed approach achieves efficient convergence even under complex model structures.

### 6.1 Setting 1: with treatment covariate only

In the simplest simulation, a single intervention covariate is used, where half of the respondents receive the intervention. The *Q* matrix is specified such that 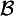 contains active parameters of 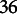 main effects without any interaction effect, as shown in Section 3.1 of the Supplementary Material. Intervention here is distributed evenly amongst the total of 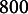 respondents, with both the control and treatment groups having a size of 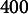 . For computational ease and at no cost to the results, the intervention treatment is applied only to the 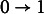 transition.

**Figure 13:**
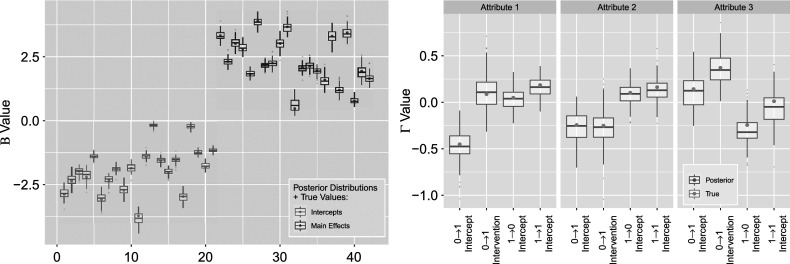
*Posterior* 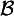 *(left) and* 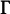 *(right) distributions bound by two standard deviations for the* 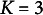 *simulation setting.* *Note*: The four indices for each attribute denote transition 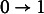 intercept, 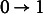 treatment (single group), 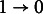 intercept, and 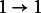 intercept, respectively.

**Figure 14:**
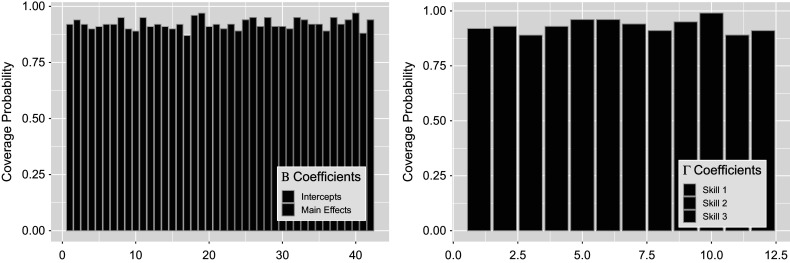
*True value coverage rates for* 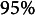 *credible intervals of* 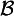 *(left) and* 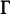 *(right) distributions bound for the* 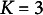 *simulation setting.* *Note*: The four indices for each attribute denote transition 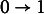 intercept, 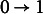 treatment (single group), 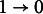 intercept, and 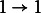 intercept, respectively.

The resulting posterior point estimates and variation bound of two standard deviations can be seen in Figure 13 for 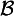 (left) and 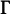 (right). For 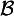 indices, the first 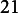 indicate intercepts and are followed by the 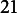 main effects. Transition 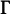 ’s are divided into three sections, corresponding to 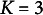 multinomial logistic regressions for each attribute. For each attribute, the transition types are indexed by 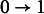 intercept, 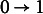 main effect, 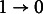 intercept, and 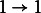 intercept, respectively. Here, the “true values” of both sets of parameters used to simulate the data modeled are also given by the solid blue and gold points for 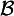 and teal points for 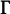 to validate our results directly. We see that the coverage of the estimated posteriors in Figure 14 indicate high rate of recovery for both sets of parameters, with an average of 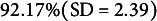 for the left figure 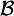 and 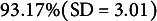 for the right figure 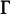 . That is, almost all of the 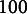 simulations produced posteriors that covered true parameter values.

This simulation setting is closely related to the empirical set-up of Section 5, and serves as the foundational case of these simulation studies. Complexity is then further added to explore how the current efficacy persists.

### 6.2 Setting 2: with item-attribute interaction terms

The following simulation setting serves to additionally include LCDM item-attribute interaction terms to the structure of 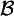 . This is done by updating the *Q* matrix to have questions impacted by multiple attributes, as shown in Section 3.1 of the Supplementary Material. Recall in Section 4.1.2 the 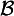 posteriors are truncated such that 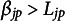 for main item-attribute effects, but not intercept effects. Interaction terms included in this study are treated the same as intercepts and are not truncated in the posterior since having full normal posteriors does not violate the monotonicity condition.

**Figure 15:**
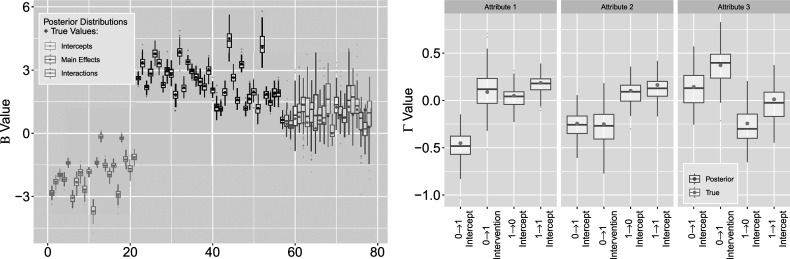
*Posterior* 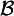 *(left) and* 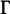 *(right) distributions bound by two standard deviations for the simulation setting with four covariates.*

**Figure 16:**
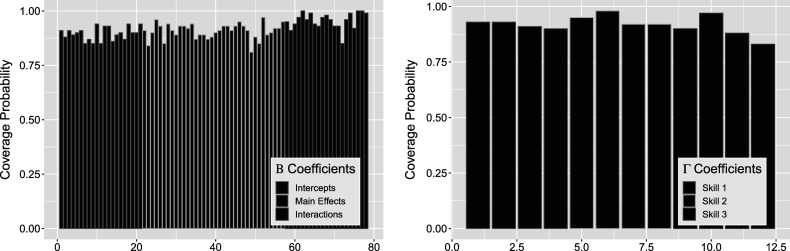
*True value coverage rates for* 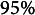 *credible intervals of* 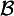 *(left) and* 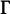 *(right) distributions bound for the* 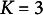 *with additional covariates simulation setting.* *Note*: The first five estimates correspond to the 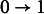 transition (intercept plus four covariate effects), followed by the next five for 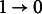 , and the intercept for 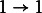 .

Figure 15 shows posterior distributions of 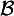 interactions in green to the right of 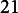 intercept terms in blue and 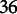 main effect terms in gold. The structure and results of 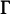 remain the same as seen in the previous Section 6.1. Notice in the figure that 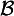 interaction terms exhibit differences compared to those of the intercepts and main effects. These interaction posteriors remain unbiased, but have large variance as the model expresses uncertainty about these estimates. Coverage of the estimated posteriors in Figure 16 again report high recovery for both sets of parameters, with an average of 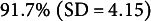 for the left figure 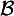 and 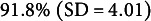 for the right figure 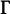 . Note that the large posterior variances on 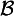 interactions have led to slightly greater coverage of 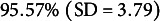 compared to other terms.

Item-attribute interactions are an aspect in most empirical settings. Although the effects themselves may not be impactful, this section shows how well the extended TDCM does with an increased number of parameters. In the following, the flexibility of the newly introduced transition regressions is updated.

### 6.3 Setting 3: with additional covariates

The inclusion of respondent covariates is a direct extension to the standard LCDM and is easily done given the updated regression formulation for LTA. This serves to be a crucial aspect for the DCMs, as individual-level covariates are nontrivial in determining how a respondent’s attribute status changes, outside the intervention treatment. The simulation setting adjustment of this section addresses this limitation of the standard TDCM and demonstrates the flexible covariate structure of 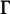 by including additional covariates.

Keeping our intervention treatment the same, we draw two variables from the uniform distribution (i.e., 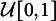 ), and the normal distribution (i.e., 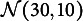 ) as toy covariates to be appended to the respondent design matrix in addition to the treatment covariate. These respondent-level covariates of the model are again only applied to the 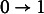 transitions for computational simplicity and ease of display.

**Figure 17:**
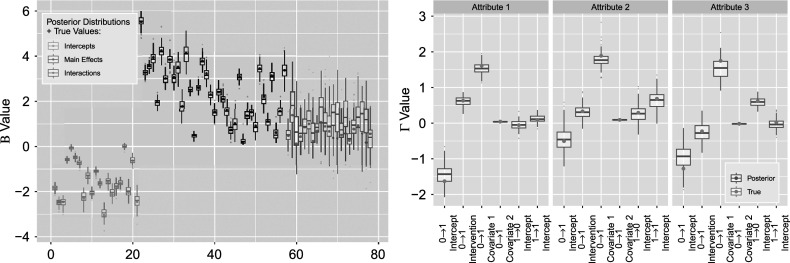
*Posterior* 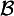 *(left) and* 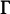 *(right) distributions bound by two standard deviations for the simulation setting with four covariates.*

**Figure 18:**
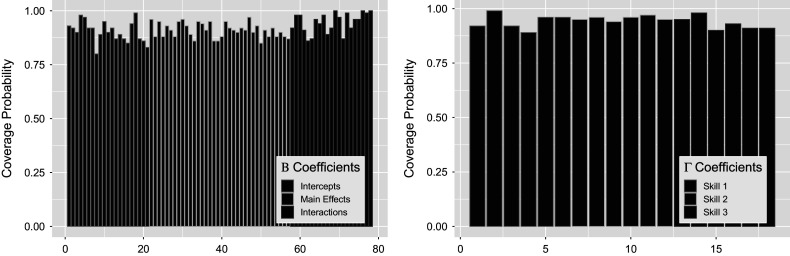
*True value coverage rates for* 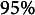 *credible intervals of* 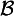 *(left) and* 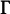 *(right) distributions bound for the* 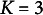 *with additional covariates simulation setting.* *Note*: The first five estimates correspond to 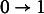 transition (intercept plus four covariate effects), followed by the next five for 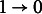 , and the intercept for 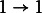 .

Figure 17 shows the estimated posteriors with the true simulation values for our two sets of parameters. Indices for 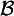 remain the same, whereas 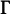 are organized such that for each attribute, the indices correspond to the 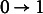 transition intercept, intervention effect, uniform distribution covariate effect, normal distribution covariate effect, followed by the intercepts for 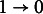 , and 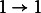 . In this right side 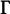 figure, we can see that the posterior distributions corresponding to the Gaussian distributed covariate (“ 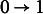 Covariate 2”) do not spread as widely as those of other covariates. This is in accordance with our expectation as the normal distribution we are drawing from has much larger mean than the other covariates, thus resulting in smaller standard error. Figure 18 finds that coverage rates for both 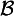 and 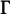 remain favorable and are not noticeably different from those of the previous simulation settings, with an average of 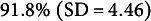 for the left figure 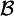 and 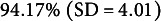 for the right figure 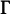 .

The results here are encouraging for the extended TDCM, as seen from the unwavering ability of the model to recover true parameter values as complexity increases. The coverage rates do not experience much, if any, change as the structural complexity as well as the number of parameters increase.

### Setting 4: 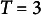 time points

6.4

We further evaluate the performance of the extended TDCM in the scenario of 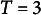 time points while keeping the remaining settings consistent with those of the previous simulations, with only the intervention treatment covariate. Note that the previously discussed empirical studies (Madison & Bradshaw, 2018a, 2018b) in Section 5 were based on two time points only. Thus, this simulation demonstrates the capacity of the extended TDCM that goes beyond the standard TDCM applications.

Here, the transition multinomial response for each logistic regression increases to 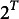 levels, or distinct ways an attribute can change between the three sequential time points. The number of responses for the multinomial regression doubles from the previous simulation and empirical settings, where 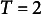 . That is, for each of the 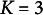 attributes, Table 2(b) shows the possible transitions ( 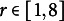 ) that can happen for 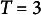 .

For three time points, assume the intervention treatment takes place between the first time point and the second time point, but not between the second and third time points. Intuitively, this is to look at the long-term effect of a single intervention occurrence. The treatment covariate is applied to the transitions 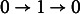 (type 3), 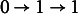 (type 4), 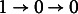 (type 5), and 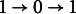 (type 6) (corresponding to 
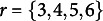
 ) in Table 2(b) while the first transition type 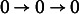 serves as the baseline constraint. Note that this setup is consistent with applying covariates to transition 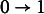 and 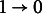 for 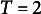 .

**Figure 19:**
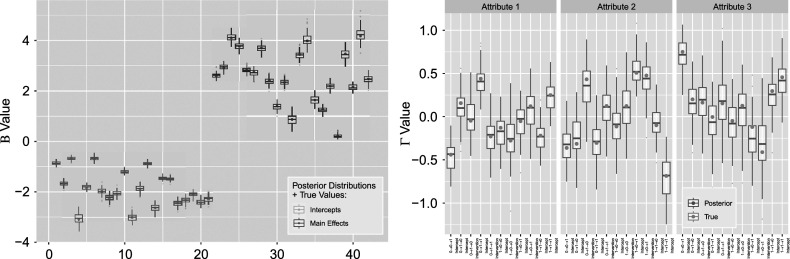
*Posterior* 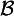 *(left) and* 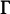 *(right) distributions bound by two standard deviations for the* 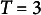 *simulation setting.*

**Figure 20:**
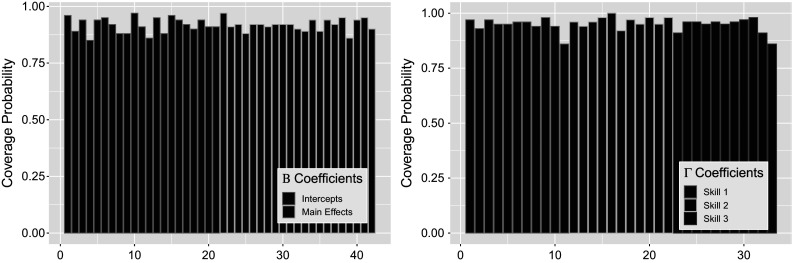
*True value coverage rates for* 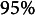 *credible intervals of* 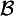 *(left) and* 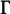 *(right) distributions bound for the* 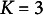 *simulation setting.* *Note*: The four indices for each attribute denote transition 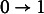 intercept, 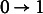 treatment (single group), 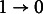 intercept, and 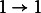 intercept, respectively.

For 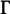 , each transition with the treatment covariate contains both an intercept and slope, while the remaining transition only has an intercept excluding the baseline transition. This totals 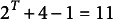 values of transition type 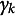 for each attribute. These 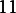 parameters for each attribute are explicitly labeled as indices in the 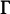 posteriors on the right side of Figure 19. The structure of 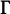 here is indexed following the order of Table 2(b). Figure 20 shows that overall the estimate intervals demonstrate proficient coverage of true values, with a mean of 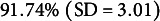 for the left figure 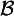 and 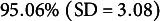 for the right figure 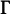 .

Useful interpretations can be inferred for specific posteriors in the figure. For example, the inclusion of intervention on students would favor the learning trajectory 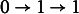 (type 4) for each of the three attributes, based on the positive intervention effect for each. This implies that the intervention would help a student who did not originally have an attribute gain the attribute in the second time point and retain it at the third time point. In addition, none of the attributes showed a positive tendency toward the 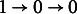 (type 5) trajectory. Naturally, the intervention here is not designed for a student who has an attribute to lose the attribute. The goal of aiding the acquisition of attributes, or at least retaining an attribute, is supported by these interpretations. We shed light on the interpretation of learning trajectories that seem less straightforward in the next section. It is also worth noting that in both of the trajectories discussed, the start state and end state are of particular interest and provide much insight. The hidden Markov DCM, however, would not allow for such interpretation due to its Markovian assumption.

## 7 Discussion

### 7.1 Summary

This study introduced a more flexible extension to the recent TDCM. The TDCM is a statistical model measuring change in latent attribute mastery over time in educational testing studies. A focus of an intervention treatment effect leads to a multiple-group context, where a treatment group receives the intervention, and a control group does not receive it. Our extension to the TDCM reformulates the standard TDCM by choosing multinomial logistic regressions to model latent transitions directly, while the LCDM item-attribute model remains the same as used for the TDCM. The primary advantage of our extension lies in the full predictive posteriors for the transition parameter 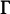 . Regression formulation allows for a full consideration of respondent-level covariates, which is not a function of the standard TDCM but an important aspect of cognitive diagnostic models. The intervention effect can then be treated as an additional covariate in implementation. Using this set-up, posteriors for attribute transition probabilities can be functionally obtained from transition 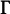 and provide a measure of the effectiveness of an intervention treatment, along with log-odds interpretability of logistic regressions.

An appealing method of Bayesian inference is crucial for a complex psychometric model such as the proposed. Whereas the standard TDCM relied on the proprietary Mplus (Muthén & Muthén, 2017), the extended TDCM offers a Gibbs sampling framework made efficient by Pòlya-gamma data augmentation. In empirical studies, we reused the mathematical education data set analyzed by the standard TDCM. Approximately same LCDM results were achieved, while the LTA regressions of the extended TDCM resulted in similar attribute transition probabilities as the standard results. In simulation studies, we considered settings to further encompass the capabilities of the extended TDCM, including additional challenges outside the scenarios explored by standard TDCM. This led to the inclusion of LCDM interaction terms, various respondent-level covariates to transition regressions, and an additional time point to demonstrate how transition regression functions in more than two time points. The results showed high coverage rates in both LCDM and transition regression parameters for each case discussed.

### 7.2 Outlook

In the current study, we assumed measurement invariance for 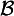 across time in all analyses. This was set for the comparisons with previous work based on the same dataset. In addition, previous studies in comparison reported no issue with the assumption. We would like to stress, though, that the proposed algorithm is flexible, and an unconstrained model without the invariance assumption can be estimated. In practice, it is recommended that users test measurement invariance before proceeding with additional analysis.

As illustrated in this article, the flexibility provided in our proposed model presents many advantages; it also serves as a point of limitation, with appropriate model specification necessary. For example, an increase in the number of time points calls for a careful choice of which transition trajectories the researcher believes matter versus which to sacrifice. The decision to model too many transition types may lead to issues with identifiability as Equation 6’s multinomial regression likelihood changes in denominator for even a few nonzero covariate slopes. This choice is also crucial to the interpretability of the model, which can gain clarity by selecting transition types that tend to be applicable to real research questions. Restricted for the standard TDCM, the proposed generalization and extension to the TDCM allows modelers to design for increased time points.

In comparison to existing longitudinal DCM models based on hidden Markov structures, the extended TDCM is able to relax the Markovian assumption that is foundational to hidden Markov DCMs. The relaxation of this assumption provides flexibility to accommodate real applications, but may lead to greater complexity for a high number of time points with poor modeling specification as discussed above. It would be of interest to compare the hidden Markov DCM with the proposed model, but an extensive comparison may be warranted in future works.

On another note, the Gibbs sampling framework of the extended TDCM alleviates some computational complexity required by standard TDCM. However, in uncommon settings, where we have a large number of time points, the parameters for transition regression increase drastically and may hinder extended TDCM’s computation performance. The flexible structure and inference method of the extended TDCM provide plentiful opportunities for future works in longitudinal DCMs.

We also acknowledge that assuming independence among the transition trajectories of different attributes can be restrictive. Considering the dependence between attributes will be a useful extension. However, it is common to have similar assumptions in previous works of literature. Li et al. (2016) proposed a LTA model that assumes the independence between attributes. Given *K* attributes and *T* time points, the number of possible learning trajectories is 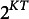 , which grows exponentially with respect to both *K* and *T*. Due to the very large number of learning trajectories and parameters, it can be very challenging to capture all those learning trajectories without enough data. Therefore, many approaches are proposed to decrease the number of learning trajectories. For example, Chen et al. (2018) suggests to only model nondecreasing learning trajectories. Similar to these ideas, we assume the independence among attributes to reduce the number of parameters to 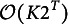 from 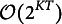 .

In conclusion, this study presented a reparametrized latent transition aspect to the TDCM and provided efficient model inference via Gibbs sampling. This extension allows for further flexibility and eases computational requirements for longitudinal (pretest/posttest) design in educational research. We are hopeful that the method we provided will assist educators in assessing growth over time and thereby better support students’ learning and improvement.

## Supporting information

Resch et al. supplementary material Resch et al. supplementary material

## Supplementary material

The supplementary material for this article can be found at https://doi.org/10.1017/psy.2025.10031.

## Appendix

### A. Derivation of posterior distributions in MCMC procedure

The notation in the appendix is the same as stated in Section 3.1. Let  be the probability density function of a Pòlya-Gamma distribution with parameter  for all  .  is the probability density function of a Pòlya-Gamma distribution with parameter  for all  .

The probability density functions or probability mass functions of  and  are listed as the following:

(A.1)

(A.2)

(A.3)

(A.4)

The joint likelihood function is:

(A.5)

(A.6)

We can now find the posterior distributions of all random variables in our model. We use  to denote the posterior distribution of *X* conditional on all other random variables except *X*. The likelihood function of  is

(A.7)

(A.8)

Therefore, we have the posterior distribution of

(A.9)

We can see that the posterior distribution of  is , where

(A.11)

(A.12)

(A.13)

(A.14)

(A.15)

(A.16)

The likelihood function of  is

(A.17)

(A.18)

(A.19)

(A.20)

where  .

The posterior distribution of  is

(A.21)

(A.22)

(A.23)

We can see that the posterior distribution of  is  , where

(A.24)

(A.25)

(A.26)

(A.27)

The posterior distribution of  follows a categorical distribution as the following:

where  is the transition of attribute *k* when  .
