# Supplementary material for: Bayesian Transition Diagnostic Classification Models with Polya-Gamma Augmentation
Source: Psychometrika. 2025 Aug 8;90(4):1368–99. doi: 10.1017/psy.2025.10031 (PMC12660026; doi:10.1017/psy.2025.10031)
Supplement: Resch et al. supplementary material [file S0033312325100318sup001.pdf]

Supplemental Material

1 Computation Times

Section	Model Specification	Runtime (min:sec)
Empirical Results		
5.1	Extended TDCM (Single Group)	9:52
5.2	Extended TDCM (Multiple Groups)	11:13
5.3	Extended TDCM (With Covariates)	10:39
Simulation Studies		
6.1	With Treatment Covariate Only	8:04
6.2	With Interaction Terms	8:11
6.3	With Additional Covariates	8:34
6.4	Three Time Points	11:53
NIMBLE Legacy Comparisons		
	Single Time Point DCM	11:01
	Standard TDCM (Single Group)	20:54

Table 10: Wall-clock runtimes (in minutes and seconds) for model estimation across all empirical and simulation settings, for one run using 3,000 MCMC iterations. Runtimes using legacy NIMBLE implementations (de Valpine et al., 2017) are included for select settings, showing the extended TDCM's computational efficiency in comparison. Computations are performed on the Apple silicon M1 processor.

2 Empirical Study

2.1 Single-Group Traceplots

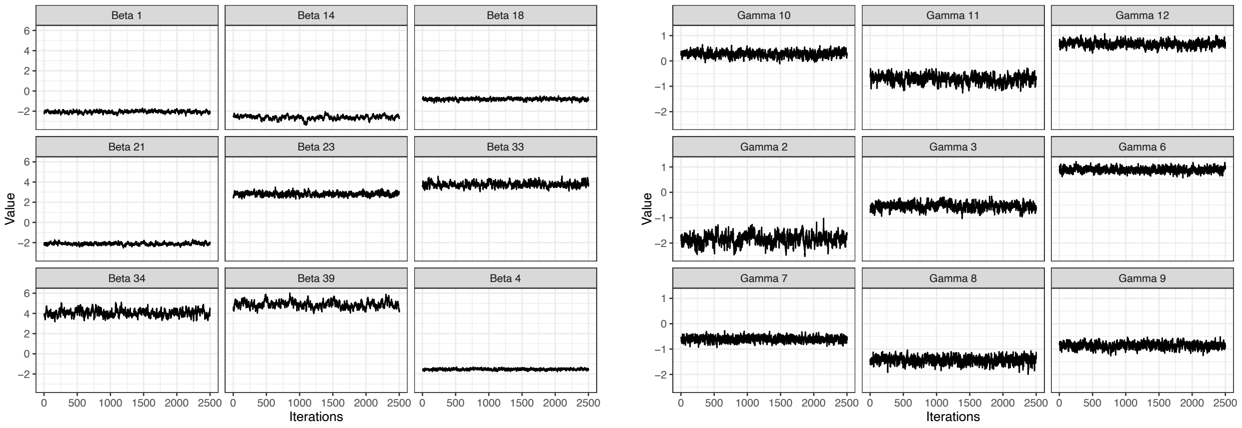

Figure 21: Trace plots of nine random parameters from $\mathcal{B}$ (left) and Γ (right) showing convergence for the single-group empirical example in Section 5.1.

2.2 Multiple-Group Traceplots

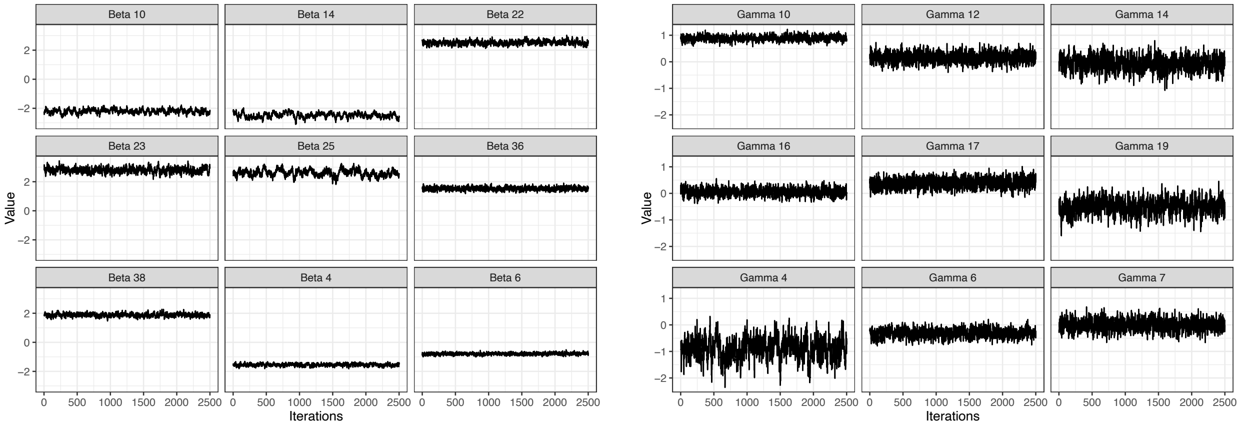

Figure 22: Trace plots of nine random parameters from $\mathcal{B}$ (left) and Γ (right) showing convergence for the multiple-group empirical example in Section 5.2.

3 Simulation Study

3.1 Q matrices

J	1	2	3	4	5	6	7	8	9	10	11	12	13	14	15	16	17	18	19	20	21
k=1	0	0	0	0	0	0	0	0	0	0	0	0	0	0	1	1	1	1	1	1	1
k=2	0	0	0	0	0	0	0	1	1	1	1	1	1	1	0	0	0	0	0	0	0
k=3	1	1	1	1	1	1	1	0	0	0	0	0	0	0	0	0	0	0	0	0	0

Table 11: Q matrix without interactions for $K = 3$ simulation data indicating attribute requirements for each $J = 21$ test questions, used in Section 6.1 and Section 6.4.

J	1	2	3	4	5	6	7	8	9	10	11	12	13	14	15	16	17	18	19	20	21
k=1	0	0	0	0	0	0	0	0	0	1	1	1	1	1	1	1	1	1	1	1	1
k=2	0	0	0	1	1	1	1	1	1	0	0	0	0	0	0	1	1	1	1	1	1
k=3	1	1	1	0	0	0	1	1	1	0	0	0	1	1	1	0	0	0	1	1	1

Table 12: Q matrix with interactions for $K = 3$ simulation data indicating attribute requirements for each $J = 21$ test questions, used in Section 6.2 and Section 6.3.

4 Simulation Setting 5: $K = 5$ with Five Covariates

To explore additional setting parameters and the extended TDCM's capabilities, we include to the simulation section a scenario with $K = 5$ attributes and five individual-level covariates over two time-points. In the previous empirical and simulation results, only $K = 3$ and $K = 4$ attributes and a maximum of two covariates were used. To compensate for the added complexity in the transition Γ's, the number of respondents has been increased to $N = 1,000$, and the number of test questions to $J = 30$. No interaction terms were included, and the Q-matrix is designed so that each question only contributes to the main effect of one particular question, as in our empirical data setting. We split the 30 questions evenly among the 5 attributes, and thus each question corresponds to 6 different questions on the test.

The covariates here are kept simple and each drawn from $\mathcal{U}[0, 1]$, and are attributed to only the $0 \to 1$ transition for computational ease and clear presentation of Γ. This elaborate setting yielded a computation time of 24 minutes and 56 seconds, more than twice the time it took for the three time point model, the previously top computationally intensive model in Section 6.4. This is reasonable given how much the addition of an attribute matters to our model.

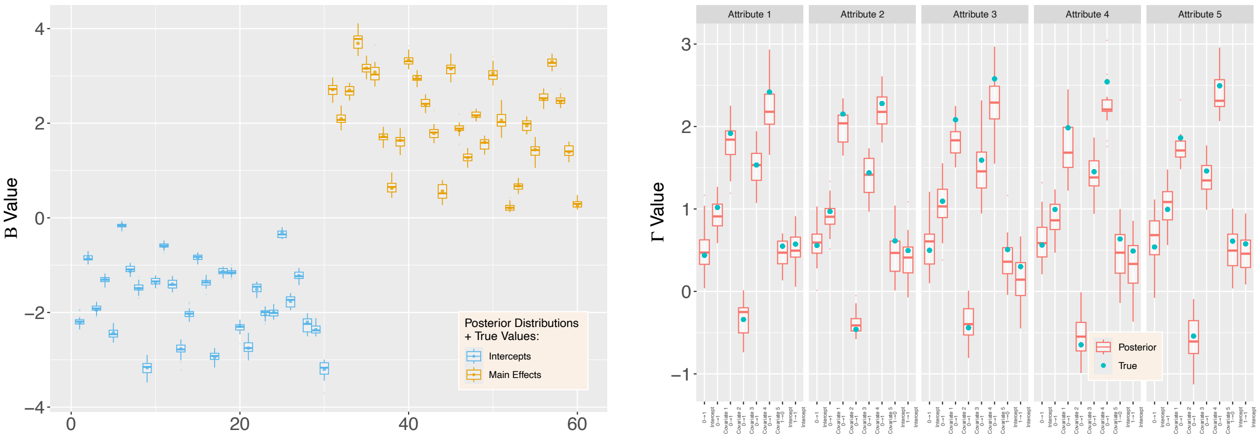

Figure 23: Posterior $\mathcal{B}$ (left) and Γ (right) distributions bound by two standard deviations for the $K = 5$ attributes and 5 covariates simulation setting.

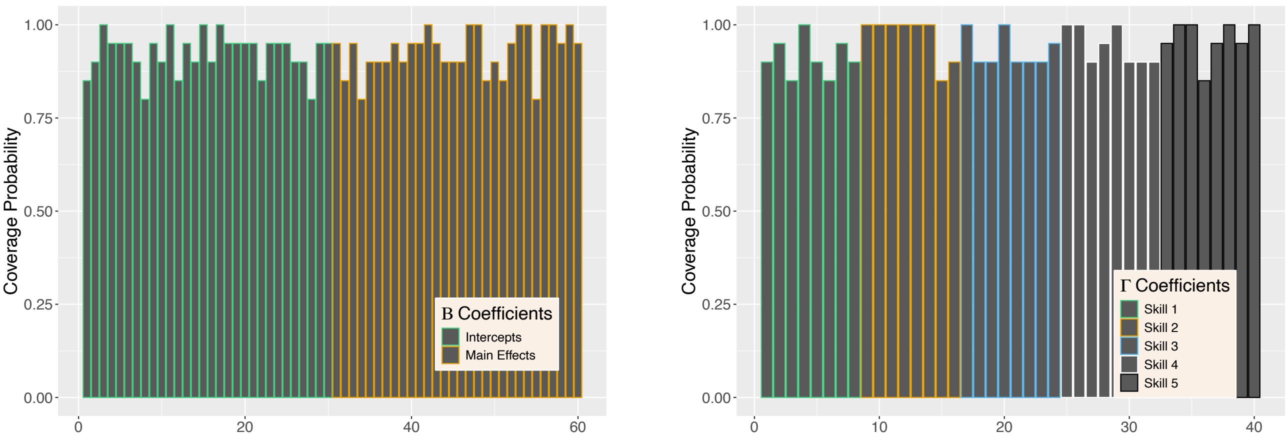

Figure 24: True value coverage rates for 95% credible intervals of $\mathcal{B}$ (left) and Γ (right) distributions bound for the $K = 3$ simulation setting. The four indices for each attribute denote transition $0 \to 1$ intercept, $0 \to 1$ treatment (single group), $1 \to 0$ intercept, and $1 \to 1$ intercept, respectively.

Results produced are consistent with those of Section 6. Figure 23 shows the estimated posteriors with the true simulation values for our two sets of parameters. Indices for $\mathcal{B}$ correspond to the interaction and main effects for the 30 test questions, whereas Γ are organized such that for each attribute, the indices correspond to the $0 \to 1$ transition intercept and the five covariate effects, followed by the intercepts for $1 \to 0$, and $1 \to 1$. We notice minor flaws in the fits here, while the posterior distributions overall show substantive evidence of reasonable fit. To further validate these results, Figure 24 finds that coverage rates to have an average of 92.75% (SD $=$ 5.63) for the left figure $\mathcal{B}$ and 94.37% (SD $=$ 5.33) for the right figure Γ. These values reflect the same

level of proficient fit as those of the previous empirical and simulation settings. Note that studies that show positive results in a setting with at least $K = 5$ attributes and five covariates are rare, but have been done with reasonable ease for the extended TDCM. Additional work on the current model class could prove to be fruitful in such complex settings.
